# Maternal Pharmaceutical Intake, Tobacco Smoking, and Environmental Pesticide Exposures: High-Resolution Serum Pesticide Screening, Longitudinal Fetal Ultrasound Biometry, and Postnatal Anogenital Distance in a Brazilian Cohort

**DOI:** 10.3390/biology15201869

**Published:** 2026-10-16

**Authors:** Ana Carolina Sater, Amanda Maieski da Silva, Ingridy Caroline de Moraes, Marco Aurelio Romano, Renata Marino Romano

**Affiliations:** Laboratory of Reproductive Toxicology and Molecular Biology, Department of Medicine, State University of Centro-Oeste (UNICENTRO), Alameda Élio Antonio Dalla Vechhia, 838, Guarapuava 85040-167, Brazil; 1712209@unicentro.edu.br (A.C.S.); 61022640009@unicentro.edu.br (A.M.d.S.); 61023140041@unicentro.edu.br (I.C.d.M.); mromano@unicentro.br (M.A.R.)

**Keywords:** prenatal ultrasound, anogenital distance, fetal biometry, tobacco smoking, pharmaceuticals, paracetamol, pesticides, suspect screening, pesticide screening, endocrine disruptors, intrauterine programming, obstetric imaging, structured questionnaire, perinatal outcomes

## Abstract

Maternal lifestyle exposures and pharmaceutical consumption during gestation represent key modulators of intrauterine growth and reproductive tract programming. This prospective cohort study evaluated the associations of maternal clinical history, medication use, and tobacco smoking with serial fetal ultrasound biometry, AGD, and postnatal infant anthropometry. A cohort of 51 pregnant women was prospectively enrolled during the first trimester (≤14 weeks) in Guarapuava, PR, Brazil, of whom 42 completed standardized longitudinal assessments across early gestation (11–14 weeks), mid-gestation (20–26 weeks), and late gestation (32–36 weeks), followed by pediatric evaluation at 8–10 months of life (*n* = 17). Clinical, sociodemographic, and pharmacological profiles were acquired through interviewer-administered structured questionnaires. Serial obstetric sonography was used to quantify the crown–rump length (CRL), estimated fetal weight (EFW), amniotic fluid index (AFI), and fetal AGD using electronic calipers. Postnatal infant AGD (anus to scrotum [AGD-AS] and anus to penis [AGD-AP] in males; anus to fourchette [AGD-AF] and anus to clitoral hood [AGD-AC] in females) was measured using digital calipers. Concurrently, high-resolution suspect pesticide screening (RP-UPLC-QTOF-MS) was performed on maternal serum collected early (≤14 weeks) and at mid-gestation (16–26 weeks) to identify circulating pesticide residues. Paracetamol was the most frequently reported drug (85.71%), followed by antibiotics (21.43%) and dipyrone (16.67%). Tobacco smoking during pregnancy was reported by 23.81% of mothers (mean: 10.10 ± 7.18 cigarettes/day). Fetal AGD expanded significantly from mid- to late gestation in both male fetuses (from 15.92 ± 3.50 mm to 31.22 ± 4.08 mm, *p* < 0.001) and female fetuses (from 12.11 ± 1.79 mm to 22.37 ± 4.46 mm, *p* < 0.001). In male infants aged 8–10 months, active maternal smoking during pregnancy was significantly associated with a marked reduction in AGD-AS (23.71 ± 3.67 mm vs. 32.89 ± 3.96 mm, *p* = 0.011) and an attenuation in AGD-AP (56.80 ± 0.99 mm vs. 71.31 ± 9.29 mm, *p* = 0.057). Exploratory analyses of therapeutic exposure to paracetamol, dipyrone, or antibiotics revealed no nominal differences in fetal biometric parameters or birth weight, although analyses were underpowered to rule out subtle effects. In female infants, birth weight correlated positively with AGD-AC (r = 0.956, *p* = 0.0435). Notably, pesticide residues were detected in 100% of the evaluated pregnant women (*n* = 42), revealing 55 distinct active xenobiotic ingredients across insecticides, herbicides, fungicides, and acaricides. The most prevalent compounds in early and mid-gestation included tebutam, pirimiphos-methyl, fenazaquin, butocarboxim, pendimethalin, silthiofam, and esprocarb. In conclusion, maternal tobacco smoking was associated with shorter male postnatal AGD in a very small subgroup (*n* = 2 exposed vs. *n* = 11 unexposed), which warrants cautious interpretation and validation in larger cohorts. High-resolution metabolomic screening provided qualitative evidence of multiclass pesticide residues in maternal circulation. Overall, this multimodal protocol illustrates a feasible surveillance framework, but the findings remain hypothesis-generating and require independent quantitative validation.

## Simple Summary

Maternal lifestyle habits and the use of common medications during pregnancy can influence intrauterine development. In this prospective clinical cohort study conducted in Paraná, Brazil, we followed pregnant women using standardized questionnaires and serial high-resolution obstetric ultrasound examinations across three trimesters, as well as pediatric assessments at 8–10 months. We observed an exploratory association between maternal tobacco smoking and shorter anogenital distance (AGD) in male infants within a small subgroup. In exploratory analyses, commonly used medications (paracetamol, dipyrone, and antibiotics) showed no detectable associations with fetal biometry or birth weight, although analyses were underpowered to exclude subtle effects. Furthermore, high-resolution serum pesticide screening qualitatively identified residues from multiple classes of pesticides in maternal serum during early and mid-gestation. These preliminary observations highlight the utility of multitiered monitoring frameworks while emphasizing the necessity of larger studies to validate potential developmental associations. These findings emphasize the clinical value of routine prenatal ultrasound combined with structured anamnesis and environmental biomonitoring to detect developmental vulnerabilities.

## 1. Introduction

Neonatal and infant health outcomes represent critical barometers of public health efficacy and socioeconomic well-being. Accumulating biological and epidemiological evidence has established that maternal physiological health, intentional pharmacological intake, and lifestyle exposures during critical embryonic and fetal development windows can induce lasting modifications in fetal tissue architecture, organ function, and hormonal programming [1,2]. Under the developmental origins of health and disease (DOHaD) paradigm, adverse intrauterine conditions or chemical insults during vulnerable developmental windows may alter developmental trajectories, predisposing offspring to reproductive, metabolic, and cardiovascular abnormalities later in life [1,2].

Among environmental and lifestyle risk factors, maternal tobacco smoking during pregnancy remains among the most widespread and detrimental exposures [3,4]. Cigarette smoke contains thousands of bioactive compounds, including nicotine, carbon monoxide, reactive oxygen species, and toxic metals such as cadmium [3,4]. Nicotine readily traverses the syncytiotrophoblast barrier and accumulates in fetal fluids and tissues [5,6]. In addition to triggering placental vasoconstriction, endothelial injury, and chronic hypoxia, tobacco compounds directly interfere with embryonic endocrine pathways [4,6,7]. Specifically, experimental and clinical studies have indicated that gestational nicotine and cotinine exposure suppresses fetal Leydig cell testosterone biosynthesis, alters steroidogenic gene expression, and modulates the androgenic milieu essential for normal male phenotypic differentiation [3,5,6].

Concurrently, the utilization of pharmaceuticals during gestation has increased globally [8]. More than 80% of pregnant women report using at least one medication to treat common obstetric complications, systemic chronic conditions, or acute symptoms such as fever, pain, and infectious episodes. Analgesics and antipyretics—predominantly paracetamol (acetaminophen) and dipyrone (metamizole)—as well as antibacterial agents, are frequently ingested during early embryogenesis and organogenesis [8,9,10,11,12]. Although paracetamol is universally positioned as a safe first-line therapeutic agent in obstetric clinical guidelines, emerging pharmacological and animal models have raised concerns regarding its inhibition of prostaglandin synthesis, cyclooxygenase (COX) pathways, and potential secondary antiandrogenic effects during the male masculinization programming window [9,10,13]. Similarly, the safety of dipyrone and broader antibiotic classes on fetal growth trajectories and endocrine endpoints requires ongoing surveillance [11,12].

In addition to intentional pharmaceutical consumption and active lifestyle habits, pregnant women residing in agricultural regions are unintentionally exposed to complex mixtures of environmental chemical contaminants, particularly agricultural and domestic pesticides [14]. Contaminants of emerging concern include agricultural pesticides, industrial chemicals, plasticizers, and surfactants that are not routinely monitored in standard antenatal care protocols [15,16]. Many of these xenobiotics act as endocrine-disrupting chemicals (EDCs), which are capable of mimicking, antagonizing, or altering endogenous hormone synthesis, transport, receptor binding, and metabolic clearance [15]. EDCs challenge traditional linear toxicological paradigms, frequently exhibiting nonmonotonic dose–response curves (such as U-shaped or inverted U-shaped profiles) and low-dose mixture effects that bypass classical regulatory safety thresholds [16]. In Brazil, acute pesticide poisoning notifications escalated dramatically between 2023 and 2026, with more than 5200 notifications recorded nationwide and more than 400 cases in the state of Paraná up to May 2026. Maternal physiological adaptations during gestation—including increased plasma volume, hyperfiltration, altered hepatic metabolism, and heightened adipose mobilization—further increase maternal–fetal vulnerability to chemical disruption, which has been associated with preeclampsia, placental dysfunction, fetal growth restriction, and developmental endocrine disorders [14,15].

In clinical perinatology, noninvasive imaging via serial high-resolution obstetric ultrasound provides an unprecedented window into intrauterine growth kinetics, anatomic development, and fetal well-being [17]. Modern sonographic protocols enable precise evaluation of fetal biometry—including crown–rump length (CRL), biparietal diameter (BPD), head circumference (HC), abdominal circumference (AC), femur length (FL), estimated fetal weight (EFW), and the amniotic fluid index (AFI) [18,19]. Beyond conventional biometry, sonographic and pediatric measurements of AGD have gained prominence as sensitive, quantitative, and sexually dimorphic anatomical biomarkers of prenatal androgen exposure [20,21,22]. During the embryonic ‘male programming window’ (8–14 weeks of gestation), testosterone and dihydrotestosterone (DHT) drive the elongation of the perineum and masculine genital development [23,24,25]. Disruptions in this signaling cascade result in shortened AGD, which serves as a clinical sentinel for impaired testicular descent and reproductive disorders [26,27,28].

Despite its clinical relevance, few prospective studies have simultaneously integrated detailed, questionnaire-verified maternal lifestyle and pharmaceutical exposure records with high-resolution serum toxicological biomonitoring of environmental pesticides, standardized longitudinal ultrasound evaluations across multiple trimesters, and paired postnatal pediatric morphometry. Therefore, the present exploratory pilot cohort study was designed to investigate maternal pharmaceutical utilization patterns, active tobacco smoking, and systemic pesticide exposure in a Brazilian obstetric cohort, evaluating preliminary correlations with longitudinal fetal ultrasound biometry, in utero AGD evolution, and postnatal infant anogenital dimensions.

## 2. Materials and Methods

### 2.1. Study Design, Setting, and Ethical Approval

**Figure 1:**
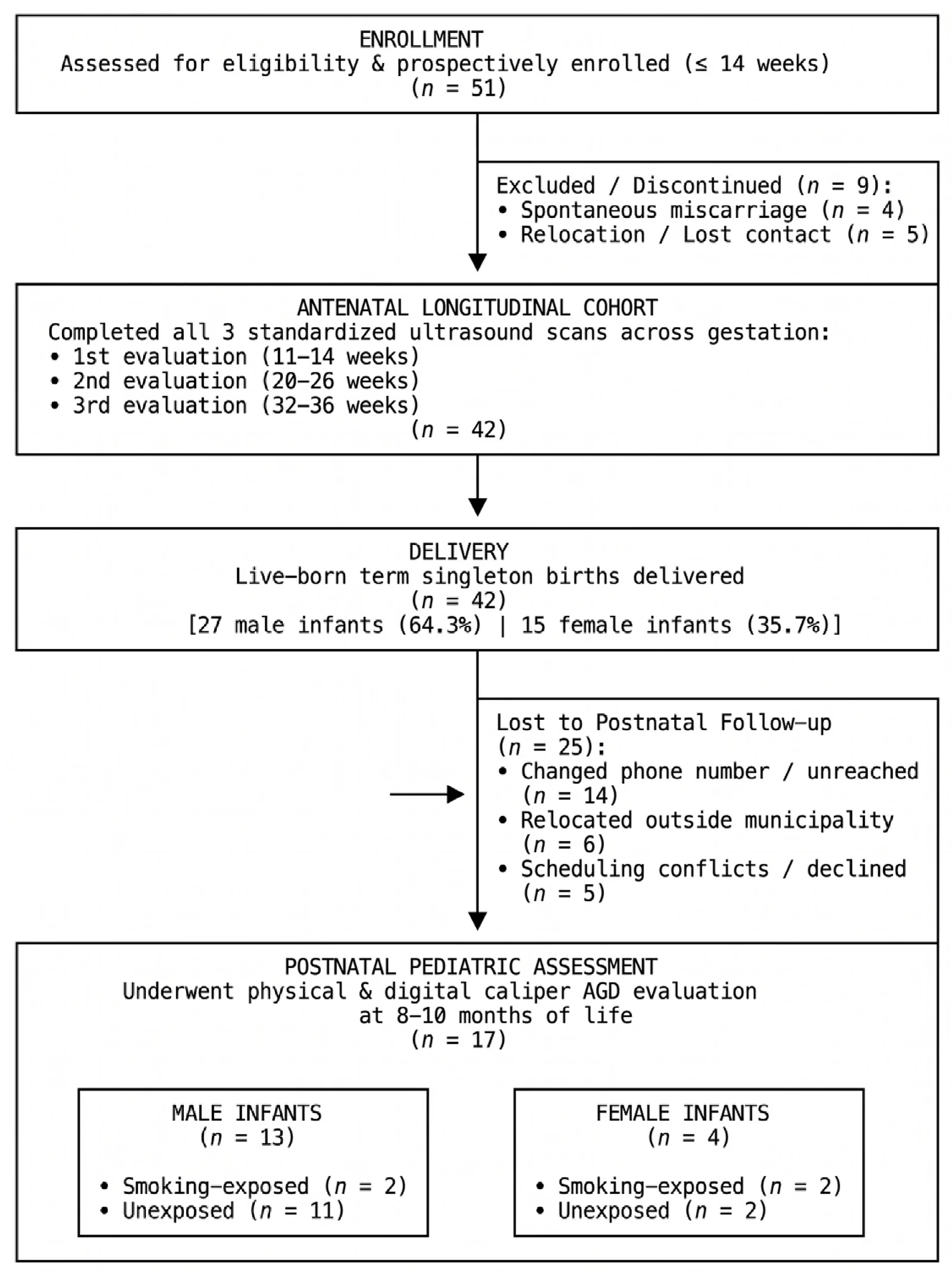
STROBE flow diagram detailing maternal enrollment, longitudinal prenatal follow-up, and postnatal pediatric evaluation.

This prospective, longitudinal exploratory pilot study was conducted at the Centro de Saúde da Mulher, a public specialized maternal–fetal healthcare reference center located in Guarapuava, Paraná, Brazil. A cohort of 51 pregnant women was consecutively enrolled during their first trimester of gestation (≤14 gestational weeks). Of these, 42 mothers remained in complete follow-up across all three designated gestational ultrasound evaluation points (early gestation: 11–14 weeks; mid-gestation: 20–26 weeks; and late gestation: 32–36 weeks) and delivered live-born singletons. A subgroup of mother–infant dyads participated in a standardized postnatal follow-up examination at 8–10 months of age (*n* = 17; 13 males, 4 females), while 25 dyads were lost to postnatal follow-up primarily because of relocation, inability to establish phone contact, or scheduling conflicts. The overall flow of participants from the initial screening through the pediatric assessment is detailed in Figure 1.

The study protocol was approved on 9 February 2023, by the Ethics Committee on Human Research of the Universidade Estadual do Centro-Oeste (UNICENTRO) under institutional certificate CAAE 65526622.7.0000.0106. The study was performed in accordance with the Declaration of Helsinki and Brazilian National Health Council (CNS) Resolution 466/2012. Written informed consent was obtained from all participants prior to study inclusion.

### 2.2. Structured Maternal Questionnaire and Clinical Data Acquisition

At study enrollment and updated during each trimester follow-up consultation, pregnant women underwent comprehensive clinical interviews utilizing a pretested, structured epidemiological questionnaire administered by trained healthcare professionals. The questionnaire systematically captured data across five functional domains:

1. Sociodemographic profile: Maternal chronological age, marital status (married, single, stable union), education level (primary, secondary, higher education completed or incomplete), and residential background.
2. Obstetric History and Clinical Comorbidities: Gravidity, parity (primiparous vs. multiparous), history of spontaneous miscarriage, pregestational maternal body mass index (BMI, categorized into normal, overweight, and pregestational obesity [BMI ≥ 30 kg/m^2^]), preexisting chronic arterial hypertension, gestational diabetes mellitus (GDM), thyroid dysfunction (hypothyroidism), and infectious episodes (gestational syphilis, candidiasis, bacterial vaginosis). The total number of prenatal consultations was recorded from official maternal antenatal health cards.
3. Tobacco and Lifestyle Exposure: A detailed history of active tobacco smoking throughout pregnancy, including smoking status (active smoker vs. nonsmoker), age of initiation, mean daily consumption (cigarettes per day), gestational trimester of exposure, and active household tobacco smoke exposure, was provided. Alcohol intake (frequency and unit quantification) and recreational substance use were similarly registered.
4. Pharmaceutical Inventory: Prospective audit of all prescription, over-the-counter (OTC), and hospital-administered medications utilized from conception through delivery. Drug identification was corroborated through physical review of medical prescriptions, drug packaging, and official maternal records. Recorded therapeutic classes included antipyretics and analgesics (paracetamol, dipyrone), nonsteroidal anti-inflammatory drugs (NSAIDs: ibuprofen, diclofenac, acetylsalicylic acid), antibacterial agents (amoxicillin, cephalosporins, macrolides, spiramycin), antihypertensive agents (methyldopa), antiemetics (metoclopramide, dimenhydrinate), antispasmodics (scopolamine), endocrine therapeutics (levothyroxine, micronized progesterone), antihistamines (loratadine), and neuropsychiatric therapeutics (sertraline, levomepromazine).
5. Perinatal and delivery records: Mode of delivery (vaginal vs. cesarean delivery), gestational age at birth (determined by first-trimester ultrasound (CRL)), birth weight (grams), infant birth length, neonatal sex, and APGAR score.

### 2.3. Longitudinal Obstetric Ultrasound Biometry and Fetal AGD Protocol

Serial transabdominal ultrasound evaluations were conducted at three standardized milestones using a high-resolution Mindray DC-7 diagnostic ultrasound system (Shenzhen Mindray Biomedical Electronics Co., Shenzhen, China) equipped with a multifrequency 4.0–8.0 MHz convex abdominal transducer. All examinations were carried out by a certified specialist in fetal medicine following standardized international criteria (ISUOG):

- First evaluation (11 + 0 to 13 + 6 gestational weeks): Validation of accurate gestational age via measurement of the fetal crown–rump length (CRL) in a midsagittal neutral plane. Structural fetal anatomy and nuchal translucency were examined.
- Second evaluation (20 + 0 to 26 + 0 gestational weeks): Second-trimester anatomical survey, fetal biometric parameters (biparietal diameter [BPD], head circumference [HC], abdominal circumference [AC], and femur length [FL]), calculation of estimated fetal weight (EFW) utilizing the Hadlock formula [18], and amniotic fluid volume quantification via four-quadrant and phenotypic genital sex determination. The amniotic fluid index (AFI) was evaluated according to standardized clinical criteria: oligohydramnios was defined as an AFI < 5 cm, normal volume as 5–24 cm, and polyhydramnios as an AFI > 24 cm [19].
- Third evaluation (32 + 0 to 36 + 0 gestational weeks): Third-trimester growth surveillance, repeat complete fetal biometry (BPD, HC, AC, FL), EFW trajectory, AFI assessment, and placental morphological characterization.

**Figure 2:**
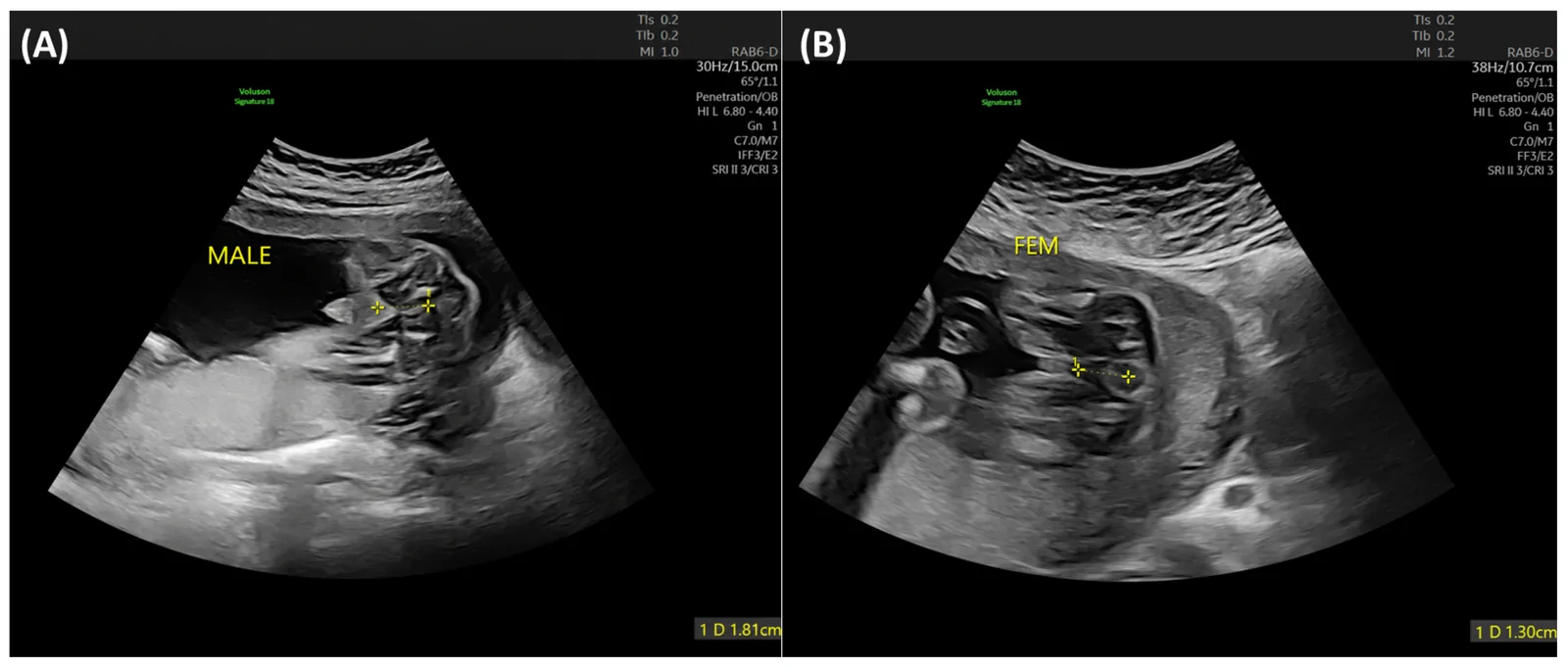
Representative transabdominal ultrasound images showing the acquisition plane for determining the fetal anogenital distance (AGD) during mid-gestation (23 weeks). (**A**) Male fetus in a tangential pelvic plane, demonstrating electronic caliper placement from the center of the anal verge to the posterior base of the scrotal sac. (**B**) Female fetus, displaying caliper placement from the anal verge to the posterior convergence of the fourchette. 1 represents the measure of AGD.

Fetal AGD was systematically assessed during the second and third trimesters according to published sonographic protocols [17]. With the fetus in a quiet, nonvertex or vertex presentation allowing an unobstructed perineal view, an axial/tangential section of the fetal pelvis was acquired. Electronic digital calipers were used to measure the linear distance: in male fetuses, from the center of the anal verge to the posterior border of the scrotal sac; in female fetuses, from the center of the anal verge to the posterior convergence of the fourchette/vestibule. Each measurement was recorded in triplicate on frozen cine loops, and the mean value was documented. Representative sonographic acquisition planes, anatomical landmarks, and caliper placements for male and female fetuses are illustrated in Figure 2. All ultrasound acquisitions were performed by a single certified fetal medicine specialist; the intraobserver measurement reproducibility across triplicate cine-loop calipers demonstrated high reliability (ICC = 0.96, 95% CI: 0.93–0.98).

### 2.4. Postnatal Infant AGD and Anthropometric Evaluation

At 8–10.5 months of postnatal age (target window: 8–10 months; mean: 9.79 ± 0.51 months, range: 8.5–10.5 months), infant anthropometry and AGD were evaluated in the pediatric outpatient service (*n* = 17; 13 males, 4 females). Examinations were performed with the infant in a relaxed supine position with the hips flexed in the “frog-leg” position by an experienced examiner. Measurements were obtained using a high-precision digital caliper (0.01 mm resolution; Mitutoyo Corp., Kawasaki, Japan) in triplicate:

- Male Infants: (1) Anus to the posterior base of the scrotum (AGD-AS); (2) Anus to the anterior base of the penis (AGD-AP); and (3) Penile width (PW) measured across the penile midshaft in a flaccid state using digital calipers placed perpendicularly to the longitudinal axis without compressing surrounding soft tissue.
- Female Infants: (1) Anus to the posterior fourchette (AGD-AF) and (2) Anus to the anterior base of the clitoral hood (AGD-AC).

Infant recumbent length was determined on a standardized infantometer (0.1 cm precision), and nude body weight was recorded on a calibrated digital pediatric scale (10 g precision). Chronological infant age at examination was documented in completed months.

### 2.5. Maternal Sample Preparation and Pesticide Screening by High-Resolution Mass Spectrometry

Maternal peripheral blood samples were collected on the same days as obstetric ultrasound examinations during early gestation (≤14 weeks) and mid-gestation (16–26 weeks). Blood samples were collected into clot-activator tubes and centrifuged at 10,000× *g* for 10 min at 4 °C to obtain serum. The serum was immediately aliquoted and stored at −80 °C at the Laboratory of Toxicology and Molecular Biology at UNICENTRO until instrumental analysis.

Pesticide screening was performed in technical collaboration with Waters Corporation (USA) using an ACQUITY UPLC I-Class liquid chromatography system coupled to a Xevo G3 QToF high-resolution mass spectrometer (Waters Corp., Milford, MA, USA). Sample preparation followed a protein precipitation procedure adapted for biological fluids. Serum aliquots were thawed on crushed ice at 4–10 °C, and a pooled quality control (QC) sample was prepared by combining 20 µL aliquots from each participant’s serum sample. For each individual sample and the pooled QC, 40 µL of serum was mixed with 20 µL of ultrapure water and 120 µL of cold acetonitrile. The mixture was homogenized for 10 min and incubated at 20 °C for 1 h to promote protein precipitation. The samples were subsequently centrifuged at 4500× *g* for 10 min at 4 °C. An 80 µL aliquot of the supernatant was transferred and concentrated to dryness using a nonheated vacuum rotary concentrator (RVC 2-18; Christ, Osterode am Harz, Germany). A sample blank was prepared under identical conditions, but serum was not added to the tube.

Chromatographic separation was performed using an ACQUITY UPLC BEH C18 analytical column (2.1 × 100 mm, 1.7 µm particle size; Waters Corp., Milford, MA, USA) maintained at 45 °C. The total run time was 15 min, with a flow rate of 0.45 mL/min and an injection volume of 5 µL. Mobile phase A consisted of 10 mM ammonium acetate in water, whereas mobile phase B consisted of 10 mM ammonium acetate in methanol. The binary gradient was programmed as follows: 0.00–0.25 min, 2% B; 0.25–12.25 min, a linear increase from 2% to 99% B; 12.25–13.00 min, 99% B; 13.00–13.10 min, a return to 2% B; and 13.10–15.00 min, column re-equilibration at 2% B.

Mass spectrometric acquisition for the reported screening results was performed in positive electrospray ionization mode (ESI+). The source parameters were as follows: capillary voltage, 1.0 kV; sampling cone voltage, 20 V; source temperature, 120 °C; desolvation temperature, 550 °C; and nitrogen desolvation gas flow, 1000 L/h. Leucine-enkephalin ([M + H]^+^ = 556.2766 *m*/*z*) was used as the reference mass calibrant. The acquisition mass range was *m*/*z* 50–1200, with a spectral acquisition rate of 4 spectra/s. The low-energy collision voltage was set to 4 eV, and the elevated-energy collision voltage was ramped from 10 to 45 eV.

Before sample analysis, a pesticide standard mixture (20 Pesticide Mix Standard, # 186006348, Waters Corp., Milford, MA, USA) containing 10 μg/mL each of acephate, atrazine, atrazine-desethyl, buprofenzin, chlortoluron, cyanazine, dicrotophos, diuron, fenpropimorph, hexazinone, linuron, methamidophos, methomyl, metobromuron, metolachlor, metoxuron, monolinuron, sebuthylazine, simazine, and terbuthylazine was diluted 100:1 in mobile phase A and injected to assess system suitability. The standard mixture was processed using the Waters UNIFI Scientific Information System before the serum samples to evaluate mass measurement errors and retention time deviations. Data processing and pesticide screening were performed using Waters UNIFI software Version 1.0 (Waters Corp., Milford, MA, USA). The screening library was restricted to pesticide compounds. Candidate signals were evaluated using predefined identification criteria, including a signal-to-noise ratio (S/N) of ≥3, a mass measurement error of ≤5 ppm, agreement between the observed and library reference retention times, and the identification of at least one fragment ion. Retention-time reference information was obtained from the UNIFI library. The pesticide standard mixture was processed before the study samples to establish reference information for screening and data evaluation.

The pooled QC sample was injected after every eight sample injections throughout the analytical sequence to monitor analytical performance. The analytical workflow and reporting of the experimental procedures were organized in accordance with the principles of transparent and reproducible chemical analysis recommended by Sumner et al. (2007) [29]. Given the screening-based identification strategy, candidate compounds were interpreted according to the available analytical evidence, without implying unequivocal structural confirmation or validated quantitative determination, using the identification–confidence framework proposed by Schymanski et al. (2014) [30] (Supplementary Material).

### 2.6. Statistical Analysis

Data management and statistical calculations were conducted using SPSS Statistics (version 27.0; IBM Corp., Armonk, NY, USA) and GraphPad Prism (version 10.4; GraphPad Software, San Diego, CA, USA). Continuous data distribution was examined for normality using the Shapiro–Wilk and Kolmogorov–Smirnov tests. Parametric variables are expressed as the mean ± standard deviation (SD), while nonnormally distributed data are presented as the median and interquartile range (IQR). Longitudinal changes in fetal AGD between the second and third trimesters were analyzed separately for male and female fetuses among complete pairs. Paired Student’s t tests and Wilcoxon signed-rank tests were applied. To account for individual variations in gestational age at each scan, the individual rate of AGD increase per gestational week was calculated for each fetus as the difference between measurements divided by the elapsed interval in exact decimal weeks. The mean weekly rates of increase with 95% confidence intervals were summarized by fetal sex. To evaluate longitudinal biometric changes across gestation, linear mixed-effects models with random subject intercepts were applied, incorporating exact gestational age at each scan as a continuous covariate. Differences between exposure groups (tobacco smokers vs. nonsmokers; pharmaceutical-exposed vs. unexposed) were evaluated using independent Student’s t tests or Mann–Whitney U tests as appropriate. Bivariate associations between birth weight, first-trimester CRL, and postnatal AGD dimensions were assessed using Pearson’s correlation coefficient (r) with 95% confidence intervals (CIs). Categorical variables were analyzed using Fisher’s exact test. A two-tailed *p* value < 0.05 was established as the criterion for statistical significance. Owing to the limited sample size in the postnatal follow-up cohort (*n* = 17), multivariable regression modeling to adjust for potential confounding variables was mathematically unfeasible without introducing severe model overfitting. Consequently, postnatal AGD comparisons are reported as unadjusted bivariate analyses and should be interpreted as strictly exploratory. The primary outcome was defined a priori as the difference in postnatal male AGD-AS between exposure groups. All other maternal, fetal biometric, and secondary subgroup analyses were exploratory; to control for multiplicity across exploratory tests, false discovery rate (FDR) adjustments using the Benjamini–Hochberg procedure were applied, with statistical significance set at an FDR-adjusted q < 0.05 (nominal *p* < 0.05).

## 3. Results

### 3.1. Maternal Sociodemographic Profile and Clinical History (Questionnaire Data)

A total of 42 pregnant women completed the prospective longitudinal protocol. Maternal sociodemographic and clinical variables obtained from the structured questionnaire are presented in Table 1. The mean age of the maternal cohort was 25.64 ± 4.63 years (range: 18 to 36 years). With respect to formal education, 50.0% had completed secondary school or higher education. Most of the participants were in a stable union (47.62%) or married (30.95%). Primiparous women accounted for 42.86% of the cohort, and 21.43% reported a prior spontaneous miscarriage. Deliveries were evenly split between vaginal birth (50.0%) and cesarean delivery (50.0%). The antenatal surveillance was rigorous, with an average of 10.64 ± 2.94 prenatal care consultations per woman.

Among clinical conditions, 19.05% of mothers had preexisting chronic arterial hypertension, and 14.29% had pregestational obesity (BMI ≥ 30 kg/m^2^). Gestational diabetes mellitus and hypothyroidism were documented in 9.52% of women each. Lower genital tract infections diagnosed during prenatal care included vaginal candidiasis (26.19%) and bacterial vaginosis (9.52%). A single participant (2.38%) was treated for gestational syphilis. To evaluate potential selection bias resulting from postnatal attrition, baseline characteristics were compared between mother–infant dyads who completed the 8–10-month pediatric follow-up (*n* = 17) and those who did not (*n* = 25). No statistically significant differences were observed between groups in terms of maternal age, active smoking during pregnancy, paracetamol exposure, gestational age at birth, or fetal sex distribution (Table 1), demonstrating that the retained postnatal subsample was representative of the initial antenatal cohort.

### 3.2. Pharmaceutical Exposure Patterns During Gestation

Systematic inquiry of medication usage revealed high rates of pharmaceutical exposure during gestation (Table 2). Paracetamol (acetaminophen) was by far the most ingested active ingredient, reported by 85.71% (*n* = 36) of women for pain or fever. Antibacterial therapeutics were prescribed to 21.43% (*n* = 9) of mothers, primarily for lower urinary tract infections and respiratory conditions. Dipyrone (metamizole) was utilized by 16.67% (*n* = 7) of the participants. Other analgesics and NSAIDs included ibuprofen (11.90%, *n* = 5), diclofenac (7.14%, *n* = 3), and acetylsalicylic acid (4.76%, *n* = 2).

Chronic medical maintenance therapy included methyldopa for hypertensive disorders (11.90%, *n* = 5) and levothyroxine for thyroid disease (9.52%, *n* = 4). Symptomatic management of gestational emesis was documented with antiemetics (11.90%, *n* = 5) and antispasmodics (scopolamine, 9.52%, *n* = 4). Psychotropic therapy was reported by two mothers receiving sertraline (4.76%) and one receiving levomepromazine (2.38%). Progestogenic luteal support (micronized progesterone) was prescribed in 4.76% (*n* = 2) of the patients.

### 3.3. Longitudinal Fetal Ultrasound Surveillance, Biometry, and Perinatal Outcomes

Longitudinal obstetric ultrasound assessments tracked fetal somatic development across trimesters (Table 3). First-trimester ultrasound confirmed gestational viability, with a mean crown–rump length (CRL) of 67.69 ± 9.34 mm at 11–14 gestational weeks. Phenotypic genital differentiation on second-trimester sonography revealed 64.29% (*n* = 27) male and 35.71% (*n* = 15) female fetuses. At the second evaluation (mean: 23.31 ± 1.96 weeks), the estimated fetal weight (EFW) was 618.38 ± 202.70 g. At the third evaluation (mean: 34.31 ± 1.28 weeks), the EFW increased to 2363.21 ± 338.96 g.

Third-trimester amniotic fluid assessment revealed a normal amniotic fluid index (AFI 5.0–24.0 cm) in 97.62% (*n* = 41) of pregnancies, with borderline-increased fluid (AFI 16.1–24.0 cm) observed in 21.42% (*n* = 9). No polyhydramnios (AFI > 24.0 cm, SMFM criteria) was diagnosed, while oligohydramnios (AFI < 5.0 cm) occurred in a single pregnancy (2.38%). At birth, the mean gestational age of the overall cohort was 39.11 ± 1.32 weeks, with a mean birth weight of 3090.43 ± 448.70 g. All the infants were born at term (≥37 weeks) without major structural malformations.

Comparative analysis of fetal biometric growth and neonatal birth weight between mothers who were exposed and those who were not exposed to major pharmaceuticals revealed no statistically significant differences. Specifically, neonates exposed to paracetamol in utero presented a birth weight of 3130.36 ± 450.02 g versus 2850.83 ± 391.00 g in unexposed neonates (*p* = 0.160). Exposure to prenatal antibiotics yielded a birth weight of 3124.33 ± 359.50 g versus 3081.18 ± 474.59 g (*p* = 0.802). Similarly, among the unexposed mothers, mothers taking dipyrone gave birth to infants weighing 3273.29 ± 391.67 g versus 3053.86 ± 455.46 g (*p* = 0.242).

### 3.4. Fetal and Postnatal AGD Trajectory

**Figure 3:**
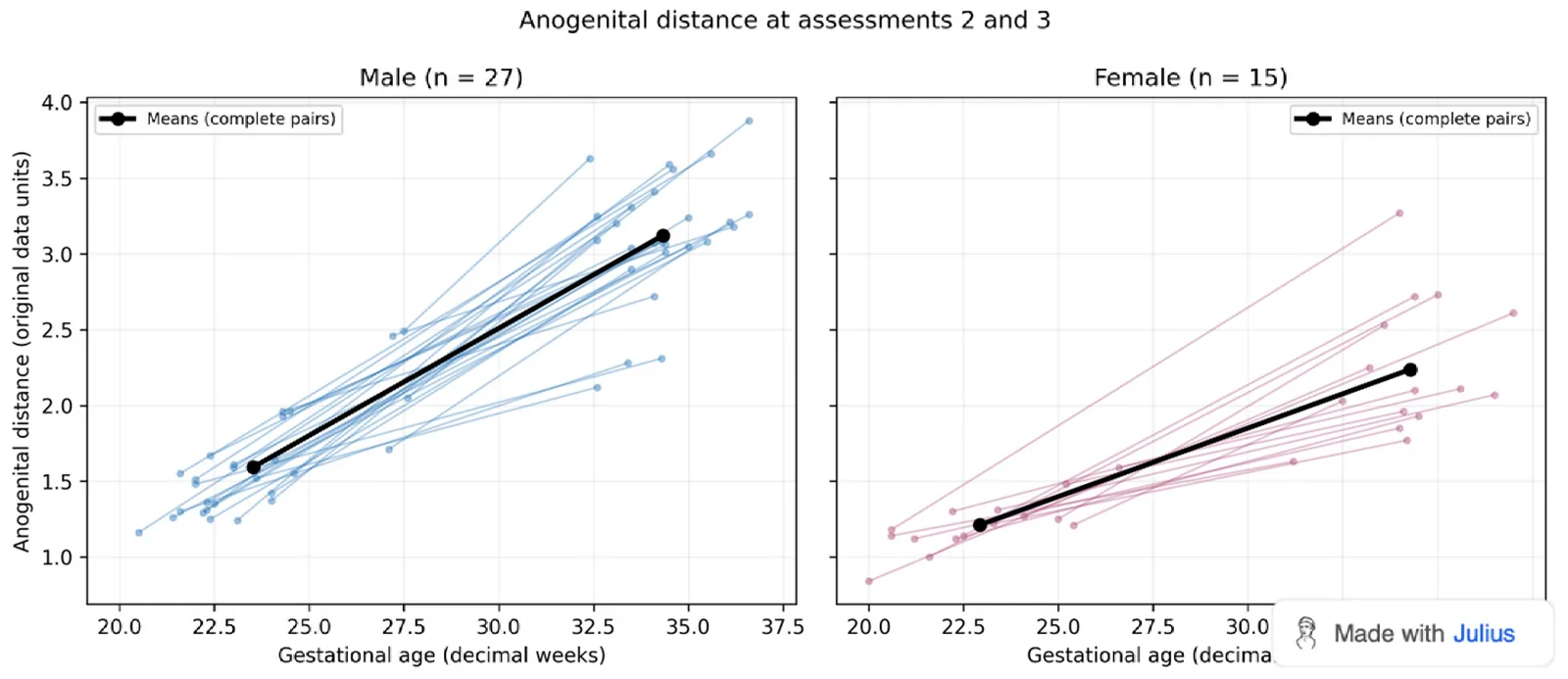
Longitudinal fetal AGD between second- and third-trimester evaluations according to exact gestational age, stratified by fetal sex. The colored lines connect paired measurements from individual fetuses across examinations. The black lines indicate the mean gestational age and mean AGD at each assessment calculated from complete pairs (males, *n* = 27; females, *n* = 15). Gestational age is expressed in decimal weeks. The figure was created by the authors using Julius AI based on data from this study.

Fetal AGD expanded significantly during intrauterine gestation across both sexes (Figure 3). In male fetuses (*n* = 27 complete pairs), fetal AGD progressed from 15.92 ± 3.50 mm at 23.52 ± 1.94 gestational weeks to 31.22 ± 4.08 mm at 34.33 ± 1.21 gestational weeks (paired t test, *p* < 0.001; Wilcoxon test, *p* < 0.001), representing an average increase of 0.144 cm per gestational week (95% CI: 0.126–0.162). In female fetuses (*n* = 15 complete pairs), fetal AGD expanded from 1.211 ± 0.179 cm at 22.93 ± 2.00 gestational weeks to 2.237 ± 0.446 cm at 34.28 ± 1.44 gestational weeks (Wilcoxon test, *p* < 0.001), reflecting an average increase of 0.090 cm per gestational week (95% CI: 0.068–0.112).

At the 8–10-month pediatric follow-up (*n* = 17; 13 males, 4 females), digital caliper morphometric measurements were successfully acquired (Table 4). In male infants, the mean anus-to-scrotum distance (AGD-AS) was 31.48 ± 5.11 mm, the anus-to-penis distance (AGD-AP) was 69.08 ± 10.08 mm, and the penile width (PW) was 11.20 ± 1.50 mm. In female infants, the mean anus-to-fourchette distance (AGD-AF) was 21.04 ± 5.25 mm, and the anus-to-clitoris distance (AGD-AC) was 51.51 ± 11.36 mm. The mean infant weight at examination was 9055.29 ± 938.37 g, with an average recumbent length of 71.35 ± 3.00 cm at a mean age of 9.79 ± 0.51 months.

Maternal exposure to common therapeutic agents showed no nominal differences in fetal or infant AGD; however, these analyses were severely unbalanced (e.g., paracetamol: 36 exposed vs. 6 unexposed; antibiotics: *n* = 9; dipyrone: *n* = 7). These exploratory comparisons were underpowered to exclude modest endocrine or developmental differences.

### 3.5. Impact of Maternal Tobacco Smoking on Male AGD

Active tobacco smoking during pregnancy was reported by 23.81% (*n* = 10) of the mothers, with an average reported intensity of 10.10 ± 7.18 cigarettes/day. Stratified comparative analysis revealed a pronounced and statistically significant reduction in AGD among male infants who were exposed to maternal smoking (Table 5). In the male subgroup (*n* = 13), compared with unexposed male infants, infants born to actively smoking mothers (*n* = 2) presented significantly shorter AGD-AS (*n* = 11; mean: 23.71 ± 3.67 mm vs. 32.89 ± 3.96 mm, Student’s t test *p* = 0.011; median: 23.71 mm [IQR 21.11–26.30] vs. 32.05 mm [IQR 29.60–36.40], Mann–Whitney U test *p* = 0.026). This represents an absolute reduction of more than 9 mm (approx. 28% reduction) in the primary androgen-sensitive perineal landmark. Furthermore, the extended male perineal landmark (AGD-AP) demonstrated a strong trend toward a reduction in smoking-exposed male infants (56.80 ± 0.99 mm vs. 71.31 ± 9.29 mm, *p* = 0.057). In female infants (*n* = 4; *n* = 2 exposed vs. *n* = 2 unexposed), although AGD-AF and AGD-AC were numerically lower in offspring of smoking mothers (AGD-AF: 17.22 ± 2.43 mm vs. 24.85 ± 4.31 mm, *p* = 0.161), the small female subsample precluded statistical confirmation.

### 3.6. Correlation Between Fetal Biometry, Birth Weight, and Infant Morphometry

Bivariate correlation analyses between neonatal birth weight, first-trimester ultrasound CRL, and postnatal AGD measurements are summarized in Table 6. In female infants (*n* = 4), a statistically significant positive correlation was demonstrated between neonatal birth weight and AGD-AC (Pearson r = 0.956, 95% CI −0.05 to 0.99, *p* = 0.0435). A strong positive trend was likewise observed for AGD-AF (r = 0.941, *p* = 0.0590). Conversely, in male infants (*n* = 13), birth weight was not significantly correlated with AGD-AS (r = −0.182, *p* = 0.5518) or AGD-AP (r = −0.072, *p* = 0.8143). Given the limited sample size, this lack of correlation cannot definitively establish the absence of somatic scaling, although it remains compatible with the literature indicating a primary role for androgen-mediated programming.

### 3.7. High-Resolution Mass Spectrometry-Based Suspect Screening of Xenobiotics in Maternal Serum

High-resolution mass spectrometry suspect screening detected signals consistent with a range of xenobiotic compounds across the maternal cohort. Pesticide residues were identified in serum samples corresponding to 100% of the participating women (*N* = 42), spanning biological samples collected across early gestation and mid-gestation. A total of 55 distinct candidate compounds were annotated through comparison with the selected screening library (Table 7). These compounds encompassed several chemical and functional categories, including agricultural pesticides, veterinary endectocides (e.g., moxidectin), feed additives and antioxidants (e.g., ethoxyquin), plant growth regulators (e.g., trinexapac-ethyl), tobacco-related compounds (e.g., nicotine), and chemical transformation products. The candidate compounds represented a broad range of pesticide classes, including insecticides (organophosphates, carbamates, neonicotinoids, pyrethroids, and spinosyns), herbicides (dinitroanilines, thiocarbamates, triazines, acetanilides, imidazolinones, and aryloxyphenoxypropionates), fungicides (triazoles, quinone outside inhibitors [QoIs], benzimidazoles, and carboxamides), and acaricides. Other annotated compounds included plant growth regulators and macrocyclic endectocides. The compounds and their corresponding screening information are summarized in Table 7.

### 3.8. Frequency of Detection of Candidate Xenobiotic Compounds Across Early and Mid-Gestation Stages

Descriptive analyses of screening results from women evaluated during early and mid-gestation were used to characterize the occurrence of candidate xenobiotic compounds across the two sampling periods (Table 8). In early gestation, the highest frequencies of signals meeting the predefined screening criteria were observed for tebutam (*n* = 42), followed by pirimiphos-methyl (*n* = 30), fenazaquin (*n* = 19), butoxycarboxim (*n* = 17), pendimethalin (*n* = 16), silthiofam (*n* = 14), and esprocarb (*n* = 14). In the mid-gestation stage, tebutam remained the most frequently detected candidate compound (*n* = 37), followed by pirimiphos-methyl (*n* = 17), pendimethalin (*n* = 17), silthiofam (*n* = 16), esprocarb (*n* = 14), fenazaquin (*n* = 13), and butoxycarboxim (*n* = 11). These results describe the frequency of candidate xenobiotic signals meeting the applied HRMS screening criteria in maternal serum samples collected during two gestational periods. However, the screening results alone do not establish confirmed residue concentrations or the sources of maternal exposure.

## 4. Discussion

In this prospective cohort study of pregnant Brazilian women and their infants, we integrated detailed anamnesis and clinical tracking via structured questionnaires with standardized serial obstetric ultrasound biometry, untargeted serum metabolomics of environmental pesticides, and pediatric follow-up. Our principal finding is that active maternal tobacco smoking during pregnancy is strongly and significantly associated with a shortened AGD from the anus to the posterior scrotum (AGD-AS) in male infants at 8–10 months of age. Concurrently, maternal consumption of paracetamol, dipyrone, or antibiotics showed no detectable associations with fetal biometry trajectories or birth weight in this small cohort, although analyses were underpowered to exclude subtle developmental effects. Crucially, our suspect screening demonstrated ubiquitous, multichemical pesticide residues across 100% of maternal blood samples during early and mid-gestation, documenting real-world chemical mixture exposures in an agricultural center.

### 4.1. Biological Mechanisms Linking Prenatal Tobacco Exposure and Shortened Male AGD

AGD is an established, lifelong anatomical biomarker of fetal androgen action during the critical embryonic ‘male programming window’ (gestational weeks 8 to 14 in humans) [20,21]. In male fetuses, the bipotential genital tubercle and perineal swellings undergo virilization under the direct influence of dihydrotestosterone (DHT), which is converted locally from testosterone synthesized by fetal Leydig cells in response to placental human chorionic gonadotropin (hCG) and pituitary luteinizing hormone (LH) [23,25]. Complete perineal elongation, scrotal fusion, and penile elongation depend upon unhindered androgenic signaling during this narrow developmental timeframe [24,26]. A shortened AGD in male offspring serves as a noninvasive anatomical biomarker associated with altered prenatal androgen signaling, although it is not pathognomonic for a single isolated mechanism [20,22].

Our preliminary observation of shorter AGD-AS among male infants born to smoking mothers (23.71 ± 3.67 mm vs. 32.89 ± 3.96 mm, *p* = 0.011) aligns with the findings of previous prospective cohort studies [27,28]. However, because the exposed male subgroup comprised only two infants, these findings must be interpreted with extreme caution as an exploratory association rather than as conclusive evidence of endocrine disruption. Mechanistically, tobacco smoke delivers a complex cocktail of toxic xenobiotics, prominently nicotine, cotinine, carbon monoxide, polycyclic aromatic hydrocarbons, and heavy metals [3,4]. Nicotine crosses the placenta unimpeded, achieving concentrations in fetal blood and amniotic fluid that exceed maternal levels by up to 15% [5]. In vitro and rodent models have demonstrated that nicotine and cotinine suppress Leydig cell proliferation, downregulate key steroidogenic enzymes (such as CYP11A1, 3β-HSD, and CYP17A1), and inhibit testosterone synthesis [3,5]. Furthermore, maternal smoking triggers placental oxidative stress, impairs spiral artery remodeling, and increases placental 11β-HSD2 activity, resulting in both direct endocrine disruption and chronic fetal microvascular hypoxia [26,27,28]. These biologically plausible pathways provide a theoretical framework for perturbations in the male programming window and shorter perineal dimensions. However, because our study did not measure maternal or cord blood sex steroids (such as testosterone or DHT), placental histopathology, or cotinine concentrations, this endocrine-disrupting mechanism remains plausible but unproven within our cohort.

In our exploratory bivariate analyses, male AGD-AS was not significantly correlated with neonatal birth weight (r = −0.182, *p* = 0.5518). While consistent with the literature describing androgenic influence during early perineal morphogenesis, the lack of statistical significance in a small cohort (*N* = 13) cannot definitively rule out somatic scaling effects, and these findings warrant evaluation in larger prospective cohorts.

### 4.2. Intentional Pharmaceutical Exposure: Patterns and Fetal Safety Profile

Our structured questionnaire revealed that 85.71% of pregnant women consumed paracetamol (acetaminophen), while 21.43% received antibiotics and 16.67% used dipyrone (metamizole). These figures mirror global trends, designating paracetamol as the most frequently used OTC medication during pregnancy [8]. Although several recent epidemiological surveys and experimental animal models have linked prolonged prenatal paracetamol exposure to antiandrogenic effects, reproductive tract abnormalities, or premature closure of the fetal ductus arteriosus [9,10,13], our exploratory findings revealed no gross alterations in second- or third-trimester fetal biometric measurements (EFW, CRL, and AFI) or birth weight associated with the therapeutic use of paracetamol, dipyrone, or antibiotics. However, because these analyses were heavily unbalanced and underpowered (e.g., 36 paracetamol-exposed vs. 6 unexposed mothers; dipyrone, *N* = 7; antibiotics, *N* = 9), the absence of statistical significance cannot be taken as an established pharmacological safety, nor can it exclude modest endocrine or developmental disruptions.

Dipyrone remains widely prescribed across Latin America and parts of Europe, although it is restricted to other jurisdictions because of agranulocytosis concerns. While preclinical studies in rodents have suggested that metamizole might alter the progesterone-to-androgen conversion ratio [11], clinical data in pregnant humans remain scarce. While our preliminary data did not reveal gross fetal growth restriction or oligohydramnios among pregnant women taking dipyrone, the small number of exposed cases (*N* = 7) precludes firm safety claims, warranting caution and larger prospective assessments.

### 4.3. Ubiquitous Environmental Pesticide Contamination, Toxicological Pathways, and Placental Vulnerabilities

A notable finding of this study was the detection of candidate xenobiotic signals meeting the predefined HRMS screening criteria in maternal serum samples from all 42 participants. These findings indicate the widespread occurrence of signals consistent with xenobiotic compounds at the sampled time points during pregnancy, although they do not establish the presence of confirmed pesticide residues or their concentrations. The annotation of 55 distinct candidate compounds, including agricultural pesticides, veterinary agents, plant growth regulators, and other xenobiotics, highlights the chemical diversity of the compounds detected through the screening approach. This pattern is consistent with the broader exposome perspective, which recognizes that human populations may encounter multiple chemicals from diverse sources throughout life [14,31]. Importantly, because our high-resolution pesticide screening was qualitative and the pesticide burden was ubiquitous (100%), no direct statistical correlation was performed between individual chemical signals and fetal biometry or AGD endpoints. Accordingly, the mechanistic pathways discussed below are presented solely as a contextual toxicological background from the external literature rather than as demonstrated clinical effects within our cohort.

While mechanistic data from in vitro systems, cell cultures, and experimental animal models provide useful biological context for the identified chemical classes, they represent theoretical toxicological pathways rather than direct evidence of fetoplacental injury in the present human cohort (Table 9):

1. Acetylcholinesterase (AChE) Inhibitors: Organophosphates (pirimiphos-methyl, dicrotophos) and carbamates (butoxycarboxim, carbosulfan) act by phosphorylating or carbamylating the serine active site of AChE, resulting in acetylcholine accumulation and parasympathetic overstimulation. In addition to acute cholinergic signs, maternal exposure to organophosphates triggers endocrine disruption, disturbing the circulating estradiol/progesterone balance and impairing endometrial decidualization [32]. In the conceptus, organophosphates readily cross the placental syncytiotrophoblast, altering placental gene coexpression networks involved in amino acid transport and cellular growth [33] and disrupting early synaptogenesis and neuronal wiring [34].
2. Agonists of Nicotinic Receptors and Neonicotinoids: The neonicotinoid acetamiprid acts as an agonist of nicotinic acetylcholine receptors (nAChRs). Experimental data have demonstrated that acetamiprid directly affects first-trimester human trophoblasts through the overproduction of reactive oxygen species (ROS) and cytoskeletal degradation [35]. Moreover, in the developing central nervous system, gestational neonicotinoid exposure promotes persistent microglial hyperactivation, promoting sustained secretion of proinflammatory cytokines such as interleukin-1beta (IL-1β), premature neuronal apoptosis, cerebellar Purkinje cell depletion, and structural anomalies, including hydrocephalus [36,37].
3. Dinitroaniline Herbicides and Endothelial Disruption: Pendimethalin (PND), detected during early and mid-gestation, induces G1 cell cycle arrest, endoplasmic reticulum (ER) stress, mitochondrial dysfunction, and apoptotic cell death in human umbilical vein endothelial cells (HUVECs) [38]. By suppressing endothelial migration and capillary tube formation, pendimethalin acts as an antiangiogenic xenobiotic capable of impairing spiral artery remodeling and fetoplacental vascular expansion. Furthermore, pendimethalin intercalates into genomic DNA with high affinity for G-C-rich sequences, conferring genotoxic and carcinogenic risks [39,40,41].
4. Mitochondrial bioenergetic disruption: The acaricide fenazaquin (a METI quinazoline) selectively blocks complex I (NADH-ubiquinone oxidoreductase) of the mitochondrial electron transport chain [42]. This blockade collapses the mitochondrial membrane potential, depletes ATP, and induces cellular bioenergetic exhaustion. Rapidly dividing embryonic and trophoblastic cells are extraordinarily sensitive to such mitochondrial uncoupling.
5. Critical Literature Gaps: Importantly, our bibliographical analysis revealed that certain compounds detected with high frequency in maternal serum—notably tebutam, silthiofam, and esprocarb—represent significant knowledge gaps in biomedical databases. For tebutam, standard searches initially retrieved literature on butane (‘Butam’) because of database synonym indexing errors; genuine toxicological evaluations in mammalian pregnancy are virtually absent. The ubiquitous presence of these poorly characterized xenobiotics in pregnant women emphasizes the urgent need for dedicated developmental toxicity testing of currently utilized agrochemicals.

**Table 9:** Reported experimental and preclinical toxicological mechanisms of detected pesticide classes in external literature models.

Compound/Class	Preclinical/In Vitro Maternal Pathways Described	Preclinical/Experimental Fetal and Placental Models Described
Nicotine (Tobacco alkaloid/Lifestyle exposure)	Appetite suppression and basal energy expenditure elevation. Autonomic cardiovascular dysregulation; hypertension risk. Central nAChR stimulation activating reward pathways.	Transplacental crossing; accumulates in amniotic fluid up to 15% above maternal circulation. Impaired trophoblast invasion/migration; reduced amino acid transport. Fetoplacental vasoconstriction and chronic hypoperfusion (IUGR). Neuroteratogenesis: cortical thinning, dendritic rarefaction, neuronal apoptosis. Attention deficit hyperactivity disorder, cognitive deficits. Blunted hypoxia chemoreflexes (SIDS risk). Pulmonary alveolar hypoplasia; DNA methylation alterations.
Pendimethalin (Dinitroaniline herbicide)	Acute gastroduodenal injury (epigastric pain, mucosal erosions). Pancreatic PANC-1 cytotoxicity: ROS generation, SOD suppression. High-affinity binding to human serum albumin (HSA). Hepatic lipid peroxidation; antiandrogenic activity; FAS/BAX upregulation.	Direct cytotoxicity on human umbilical vein endothelial cells (HUVEC). G1 cell cycle arrest and apoptosis via ER stress and mitochondrial collapse. Disrupted endothelial migration and tube formation (anti-angiogenic). Direct DNA intercalation (G-C rich regions); Group C possible carcinogen.
Pirimiphos-methyl & Organophosphates	Systemic AChE inhibition and parasympathetic hyperactivation. Endocrine disruption: antiandrogenic effect; estradiol/progesterone imbalance. Defective blastocyst implantation and decidualization.	Transplacental crossing; dysregulation of placental gene coexpression networks. Reduced birth weight and intrauterine growth restriction. Disrupted synaptogenesis and neural circuit construction. In vitro hepatocyte mitochondrial bioenergetic dysfunction.
Acetamiprid & Neonicotinoids	Autonomic cholinergic signaling perturbation. Systemic immune modulation and oxidative stress.	First-trimester human trophoblast cytotoxicity via ROS and proteolysis. Sex-specific elevation of placental BChE activity in female fetuses. Fetal microglial overactivation, IL-1beta release, and neuroinflammation. Cortical neuron apoptosis, Purkinje cell loss, motor/cognitive deficits. Postimplantation fetal resorption, anencephaly, and hydrocephalus.
Pyrethroids (Pyrethrin I, Deltamethrin)	Severe interstitial lung disease following inhalation (dyspnea, opacities). Genotoxicity in human lymphocytes: elevated SCEs, 8-OHdG oxidative lesions.	Prolonged voltage-gated Na+ channel opening in fetal/neonatal CNS. Neuronal hyperexcitability, recurrent tonic–clonic seizures, and neonatal coma.
Fenazaquin (Quinazoline/METI)	Hepatic microsomal cytochrome P450 overload and bioenergetic stress.	Specific Complex I respiratory chain inhibition, ATP depletion, and ADP accumulation. Disruption of membrane glycerophospholipid metabolism.
Deethylatrazine & Triazines	Endocrine disruption; epidemiological associations with gestational hypertension.	Disruption of fetal HPG axis via altered LH surge; ubiquitin–proteasome inhibition.
Tebutam, Esprocarb, Silthiofam	High analytical prevalence in maternal serum; mechanistic maternal data limited.	Critical literature gap: lack of direct empirical evidence on mammalian embryotoxicity (theoretical risk).

Note: Maternal, placental, and fetal lesions reflect established toxicological mechanisms characterized in experimental animal, in vitro trophoblast, or human cell-line models cited in the text; clinical translation during low-dose human pregnancy exposures warrants direct confirmation.

### 4.4. Methodological Strengths of Longitudinal Ultrasound and Structured Questionnaires

A major strength of this study lies in the prospective, multistage longitudinal methodology. By performing transabdominal obstetric ultrasound scans at predefined intervals (11–14 weeks, 20–26 weeks, and 32–36 weeks), we were able to validate accurate embryonic dating via the CRL, objectively measure fetal biometric velocities, monitor amniotic fluid volume, and capture fetal AGD in utero. The observed progression of fetal AGD across gestation—0.144 cm/week in males and 0.090 cm/week in females—confirms the physiological expansion of the fetal pelvic and perineal structures, demonstrating the feasibility of prenatal AGD sonographic tracking.

Furthermore, conducting structured, face-to-face questionnaire audits across successive visits minimized recall bias regarding medication names, dosages, and smoking habits. Maternal self-reports were systematically corroborated through health provider records and physical prescription audits. The high average number of prenatal visits in this cohort (10.64 ± 2.94) indicates that the clinical population was engaged, reducing lost-to-follow-up bias during the pregnancy phase.

### 4.5. Study Limitations and Future Directions

Several methodological limitations must be acknowledged. First, the final analytical cohort comprised 42 completed maternal follow-ups, and the pediatric cohort at 8–10 months included 17 infants. Specifically, within the postnatal male cohort (*n* = 13), only two infants were born to mothers who actively smoked during pregnancy. Given this very small subgroup size, statistical inference is intrinsically sensitive to individual variance, precluding definitive conclusions regarding causality or mechanistic androgen receptor antagonism. Similarly, the postnatal female cohort was limited to four infants (*n* = 4); post hoc correlation analyses in this subgroup are strictly preliminary and cannot substantiate definitive physiological mechanisms of female perineal development. Given that this was designed as an exploratory pilot cohort, an a priori statistical power calculation was not performed. Although a statistically significant reduction in male AGD-AS was detected in relation to smoking (*p* = 0.011), statistical significance cannot be conflated with adequate study power; this finding is susceptible to small-sample instability and potential effect-size inflation (type M error). Furthermore, pharmaceutical exposure characterization was inherently limited. Analyses lacked granular data on cumulative dosage, duration, and trimester-specific timing (e.g., paracetamol exposure encompassed anything from a single tablet to repeated use), and the potential for confounding by the underlying medical indication could not be completely excluded.

Second, postnatal follow-up was completed by 17 out of 42 eligible dyads, representing an attrition rate of 59.5%. Although comparative analysis between retained participants and those lost to follow-up revealed no significant disparities in baseline sociodemographics, smoking rates, or drug intake, the risk of residual selection bias cannot be entirely ruled out. Loss to follow-up was largely attributable to logistical hurdles in public healthcare settings, such as telephone number changes and transportation constraints.

Third, maternal tobacco exposure was quantified via structured self-report questionnaires without corroborative biochemical assays (e.g., serum or urinary cotinine quantification). Although self-reported cigarette counts showed robust clinical associations, biochemical validation in larger cohorts would enable dose–response gradient analyses.

Fourth, while comprehensive 2D fetal biometry and AGD were captured, future studies would benefit from incorporating advanced hemodynamic indices—such as uterine, umbilical, and fetal middle cerebral artery Doppler pulsatility indices—to evaluate subtle placental resistance adaptations. Additionally, the observed weekly fetal AGD expansion reflects an average rate of change between two discrete observation points and does not assume a strictly linear trajectory across entire gestation.

Fifth, because our suspect screening was qualitative, it cannot distinguish trace analytical detections from biologically active internal exposure levels, highlighting the need for future targeted quantitative assays. Ongoing studies in our laboratory are currently systematizing these chemical compound matrices, evaluating clustering algorithms for multichemical mixtures, and establishing targeted concentration curves to correlate specific residue tiers directly with fetal biometric velocities. Finally, given the extensive exploratory biometric and metabolomic evaluations, findings beyond the primary male AGD endpoint should be interpreted cautiously as hypothesis-generating, pending confirmation in larger preregistered cohorts.

## 5. Conclusions

In conclusion, this prospective cohort study provides preliminary observations of an exploratory association between maternal tobacco smoking and shorter AGD-AS in male infants at 8–10 months of age. Given the very small exposed subgroup (*N* = 2), these findings cannot establish definitive evidence of androgen signaling disruption and warrant validation in larger cohorts. Concurrently, maternal therapeutic use of common pharmaceuticals showed no detectable associations with gross fetal biometry or birth weight, although analyses were underpowered to exclude subtle endocrine alterations. High-resolution mass spectrometry-based suspect screening revealed candidate compounds belonging to multiple pesticide classes in maternal serum samples collected during pregnancy, highlighting the diversity of xenobiotic signals observed in maternal circulation. Overall, while serial ultrasound biometry paired with exposure questionnaires and metabolomic screening represents a feasible multitiered research strategy, our findings remain hypothesis-generating; independent quantitative validation in larger, preregistered prospective cohorts is needed before clinical surveillance implications can be determined.

## Figures and Tables

**Table 1:** Maternal baseline sociodemographic characteristics, clinical history, and lifestyle factors (*N* = 42).

Variable/Parameter	Cohort Value/*n* (%)
Maternal Age (years), mean ± SD [range]	25.64 ± 4.63 [18–36]
Educational Level, *n* (%)	
Primary School (Complete)	2 (4.76%)
Primary School (Incomplete)	4 (9.52%)
Secondary School (Complete)	11 (26.19%)
Secondary School (Incomplete)	10 (23.81%)
Higher Education (Complete)	6 (14.29%)
Higher Education (Incomplete)	9 (21.43%)
Marital Status, *n* (%)	
Married	13 (30.95%)
Single	9 (21.43%)
Stable Union	20 (47.62%)
Obstetric History, *n* (%)	
Primiparous (1 gestation)	18 (42.86%)
Multiparous (>1 gestation)	24 (57.14%)
Previous Miscarriage	9 (21.43%)
Delivery Mode, *n* (%)	
Vaginal Delivery	21 (50.00%)
Cesarean Section	21 (50.00%)
Maternal Comorbidities, *n* (%)	
Chronic Systemic Arterial Hypertension	8 (19.05%)
Pregestational Obesity (BMI ≥ 30 kg/m^2^)	6 (14.29%)
Gestational Diabetes Mellitus (GDM)	4 (9.52%)
Hypothyroidism	4 (9.52%)
Gestational Syphilis	1 (2.38%)
Vaginal Candidiasis	11 (26.19%)
Bacterial Vaginosis	4 (9.52%)
Lifestyle Habits, *n* (%)	
Active Tobacco Smoking	10 (23.81%)
Cigarettes Smoked per Day, mean ± SD	10.10 ± 7.18
Alcohol Consumption	1 (2.38%)
Prenatal Care Visits, mean ± SD	10.64 ± 2.94

**Table 2:** Reported intentional pharmaceutical usage during pregnancy (*n* = 42).

Therapeutic Class/Pharmaceutical Compound	Exposed Patients, *n* (%)
Paracetamol (Acetaminophen)	36 (85.71%)
Antibiotics (Systemic, various classes)	9 (21.43%)
Dipyrone (Metamizole)	7 (16.67%)
Ibuprofen	5 (11.90%)
Methyldopa	5 (11.90%)
Antiemetics (Metoclopramide/Dimenhydrinate)	5 (11.90%)
Scopolamine (Hyoscine butylbromide)	4 (9.52%)
Levothyroxine Sodium	4 (9.52%)
Diclofenac	3 (7.14%)
Acetylsalicylic Acid (Low-dose AAS)	2 (4.76%)
Acyclovir	2 (4.76%)
Sertraline	2 (4.76%)
Micronized Progesterone	2 (4.76%)
Loratadine	2 (4.76%)
Spiramycin	1 (2.38%)
Levomepromazine	1 (2.38%)

**Table 3:** Longitudinal fetal ultrasound biometric parameters, amniotic fluid dynamics, and neonatal birth outcomes.

Fetal Ultrasound & Perinatal Parameter	Cohort Value/*n* (%)
Fetal Genital Sex, *n* (%)	
Male	27 (64.29%)
Female	15 (35.71%)
First Evaluation (11 + 0 to 13 + 6 weeks)	
Crown-Rump Length (CRL, mm), mean ± SD	67.69 ± 9.34
Second Evaluation (20 + 0 to 26 + 0 weeks)	
Gestational Age at Scan (weeks), mean ± SD	23.31 ± 1.96
Estimated Fetal Weight (EFW, g), mean ± SD	618.38 ± 202.70
Male fetal AGD (anus–scrotum, mm), mean ± SD Female fetal AGD (anus–fourchette, mm), mean ± SD	15.92 ± 3.50 12.11 ± 1.79
Third Evaluation (32 + 0 to 36 + 0 weeks)	
Gestational Age at Scan (weeks), mean ± SD	34.31 ± 1.28
Estimated Fetal Weight (EFW, g), mean ± SD	2363.21 ± 338.96
Male fetal AGD (anus–scrotum, mm), mean ± SD Female fetal AGD (anus–fourchette, mm), mean ± SD	31.22 ± 4.08 22.37 ± 4.46
Amniotic Fluid Index (AFI) Profile, *n* (%)	
Normal AFI (8.0–16.0 cm)	32 (76.19%)
Borderline/Increased AFI (16.1–24.0 cm)	9 (21.42%)
Polyhydramnios (>24.0 cm)	0 (0.00%)
Oligohydramnios (<8.0 cm)	1 (2.38%)
Neonatal Birth Outcomes	
Gestational Age at Delivery (weeks), mean ± SD	39.11 ± 1.32
Birth Weight (g), mean ± SD	3090.43 ± 448.70

**Table 4:** Postnatal infant AGD and anthropometric parameters at 8–10 months of age (*n* = 17).

Pediatric Anthropometric/AGD Endpoint	Mean ± SD [Range]
Male Infants (*n* = 13)	
AGD-AS (Anus to posterior base of scrotum, mm)	31.48 ± 5.11 [21.11–37.80]
AGD-AP (Anus to anterior base of penis, mm)	69.08 ± 10.08 [56.09–81.20]
Penile Width (PW at mid-shaft, mm)	11.20 ± 1.50 [9.10–14.00]
Female Infants (*n* = 4)	
AGD-AF (Anus to posterior fourchette, mm)	21.04 ± 5.25 [15.55–27.90]
AGD-AC (Anus to anterior base of clitoral hood, mm)	51.51 ± 11.36 [35.50–61.70]
Overall Infant Cohort Anthropometry (*n* = 17)	
Infant Body Weight (g)	9055.29 ± 938.37 [7500–10,800]
Recumbent Length (cm)	71.35 ± 3.00 [66.5–76.0]
Infant Chronologic Age (months)	9.79 ± 0.51 [8.5–10.5]

**Table 5:** Comparison of infant AGD at 8–10 months stratified by maternal tobacco smoking during pregnancy.

Infant Morphometric Endpoint	Maternal Smoking (Yes)	Maternal Smoking (No)	*p* Value
Male Infants (*n* = 13)	(*n* = 2)	(*n* = 11)	
AGD-AS (mm), Mean ± SD	23.71 ± 3.67	32.89 ± 3.96	0.011 *
AGD-AS (mm), Median (IQR)	23.71 (21.11–26.30)	32.05 (29.60–36.40)	0.026 *
AGD-AP (mm), Mean ± SD	56.80 ± 0.99	71.31 ± 9.29	0.057
AGD-AP (mm), Median (IQR)	56.80 (56.09–57.50)	74.60 (62.30–78.90)	0.103
Female Infants (*n* = 4)	(*n* = 2)	(*n* = 2)	
AGD-AF (mm), Mean ± SD	17.22 ± 2.43	24.85 ± 4.31	0.161
AGD-AF (mm), Median (IQR)	17.22 (15.55–18.94)	24.85 (21.80–27.90)	0.333
AGD-AC (mm), Mean ± SD	46.07 ± 14.94	56.95 ± 6.71	0.447
AGD-AC (mm), Median (IQR)	46.07 (35.50–56.64)	56.95 (52.20–61.70)	1.000

* Statistically significant difference (*p* < 0.05). IQR: Interquartile Range.

**Table 6:** Pearson correlation between birth weight, first-trimester CRL, and infant AGD at 8–10 months.

Parameter	AGD-AS (Male)	AGD-AP (Male)	AGD-AF (Female)	AGD-AC (Female)
Birth Weight (g)				
Pearson r	−0.182	−0.072	0.941	0.956
95% Confidence Interval	−0.66 to 0.41	−0.59 to 0.49	−0.20 to 0.99	−0.05 to 0.99
*p* value	0.5518	0.8143	0.0590	0.0435 *
First-Trimester CRL (mm)				
Pearson r	0.4487	0.1565	−0.1653	0.0588
95% Confidence Interval	−0.13 to 0.80	−0.43 to 0.65	−0.97 to 0.94	−0.95 to 0.96
*p* value	0.1240	0.6096	0.8347	0.9411

* Statistically significant correlation (*p* < 0.05). CRL: crown–rump length.

**Table 7:** Classification and characterization of candidate xenobiotic compounds annotated in maternal serum by high-resolution mass spectrometry-based suspect screening.

Xenobiotic Category/Use	Chemical Class	Proposed Mechanism/Biological Target	Chemical Compound
Insecticide	Carbamate	AChE inhibition (parasympathetic hyperstimulation)	3-Hydroxycarbofuran
			Benfuracarb
			Butoxycarboxim
			Carbosulfan
	Organophosphate		Dicrotophos
			(E)-Phosphamidon
			Pirimiphos-methyl
Insecticide	Neonicotinoid	Nicotinic acetylcholine receptor (nAChR) agonist	Acetamiprid
			Nitenpyram
Tobacco alkaloid/Lifestyle xenobiotic	Primary alkaloid		Nicotine
Insecticide	Spinosyn		Spinetoram
Insecticide	Pyrethroid	Prolonged voltage-gated Na+ channel opening	Bioresmethrin
			Deltamethrin
			Pyrethrin I
Insecticide	Semicarbazone	Voltage-gated Na+ channel blockade	Metaflumizone
	Pyrazole	Ryanodine receptor activation (muscle paralysis)	Chlorantraniliprole
	Carbohydrazide	Ecdysone receptor agonist (molting disruption)	Tebufenozide
	Azoxy	Metabolic pathway interference	Phenazox
Insecticide/Piscicide	Rotenone	Mitochondrial oxidative phosphorylation uncoupling	Rotenone
Herbicide	Thiocarbamate	Aldehyde dehydrogenase inhibition/oxidative stress	Dimepiperate
	Anilide	Very-long-chain fatty acid (VLCFA) synthesis inhibition	Pentanochlor
	Acetanilide	VLCFA inhibition/cytotoxicity	Acetochlor
	Chloroacetanilide	VLCFA synthesis inhibition	Metolachlor
	Chloroacetamide	VLCFA synthase/KCS inhibition	Metazachlor
	Thiocarbamic ester	VLCFA elongase inhibition	Esprocarb
	Urea	Photosystem II (PSII) electron transport inhibitor	Cycluron
	Urea	Photosystem II (PSII) inhibitor	Fenuron
	Triazine	PSII inhibitor/Ubiquitin–proteasome inhibition	Cyanazine
	Triazine	Atrazine metabolite/PSII inhibitor/HPG axis disruption	Deethylatrazine
	Triazine	Terbuthylazine transformation product/PSII inhibitor	Sebuthylazine-desethyl
	Triazine	PSII photosynthesis inhibitor	Secbumeton
	Benzothiazole	Photosystem II (PSII) inhibitor	Methabenzthiazuron
	Pyrrolidine	Carotenoid biosynthesis inhibition (bleaching)	Flurochloridone
	Propanamide	Acyl-ACP thioesterase (FAT) lipid synthesis inhibitor	Tebutam
	Imidazolinone	ALS/AHAS enzyme inhibitor	Imazamox
	Imidazolinone	ALS/AHAS enzyme inhibitor	Imazapyr
	Dinitroaniline	Microtubule assembly and cell division inhibition	Pendimethalin
	AOPP/FOP	Acetyl-CoA carboxylase (ACCase) inhibitor	Fluazifop-p-butyl
	CHD/DIM	Acetyl-CoA carboxylase (ACCase) inhibitor	Cycloxydim
	AOPP/FOP	Acetyl-CoA carboxylase (ACCase) inhibitor	Propaquizafop
	Pyridine	Synthetic auxin (pyridine-2-carboxylate/picolinate)	Picloram
Herbicide/Antioxidant	Quinoline	Electron donor for free radicals/redox imbalance	Ethoxyquin
Fungicide	Urea	DHFR and nucleic acid/protein synthesis inhibition	Cymoxanil
	Anilide	Target molecular site uncharacterized	Pyracarbolid
Fungicide/Regulator	Phenylurea	Fruit maturation acceleration/hormonal signaling	Forchlorfenuron
Fungicide/Biocide	1,2-Thiazole	Thiol redox alteration and mitochondrial damage	Octhilinone
Fungicide	QoI Strobilurin	Mitochondrial cytochrome b respiratory chain inhibitor	Metominostrobin
	Aminopyrimidine	Pectinase inhibition/AhR association	Mepanipyrim
	Benzimidazole	Beta-tubulin binding/microtubule assembly block	Carbendazim
	Cyclopentanol	Ergosterol 14alpha-demethylation (CYP51) inhibitor	Ipconazole
	Thiophene	Antifungal activity with unidentified mammalian site	Silthiofam
Acaricide	Organophosphate	Acetylcholinesterase (AChE) inhibition	Diazinon
	Quinazoline	Mitochondrial respiratory Complex I (METI) inhibitor	Fenazaquin
Plant Growth Reg.	Cyclohexanone	Gibberellin biosynthesis inhibitor	Trinexapac-ethyl
Endectocide	Macrocyclic lactone	GluCl channel binding with chloride influx	Moxidectin

Note: Proposed biochemical pathways and target receptors are compiled from standard agrotechnical classifications (IRAC, FRAC, HRAC modes of action) and preclinical mechanistic toxicological literature.

**Table 8:** Descriptive comparison of the most frequently detected candidate pesticide compounds in maternal serum during early and mid-gestation (*n* = 42 paired mothers).

Active Ingredient	1st Trimester Detection (*n* = 42)	2nd Trimester Detection (*n* = 42)
Tebutam	42	37
Pirimiphos-methyl	30	17
Fenazaquin	19	13
Butoxycarboxim	17	11
Pendimethalin	16	17
Silthiofam	14	16

## Data Availability

The data presented in this study are available upon reasonable request from the corresponding author because of patient privacy and institutional ethical restrictions.

## Supplementary Materials

The following supporting information can be downloaded at https://www.mdpi.com/article/10.3390/biology15201869/s1. Table S1. Analytical evidence and provisional identification confidence for the TEBUTAM candidate; Table S2. Analytical evidence and provisional identification confidence for the PIRIMIPHOS-METHYL candidate; Table S3. Analytical evidence and provisional identification confidence for the FENAZAQUIN candidate; Table S4. Analytical evidence and provisional identification confidence for the BUTOXYCARBOXIM candidate; Table S5. Analytical evidence and provisional identification confidence for the PENDIMETHALIN candidate; Table S6. Analytical evidence and provisional identification confidence for the SILTHIOFAM candidate; Table S7. Analytical evidence and provisional identification confidence for the ESPROCARB candidate; Figure S1. Chromatogram and mass spectra associated with the tentatively identified Tebutam candidate: (A) chromatogram of the [M+H]^+^ channel, showing a peak at 8.75 min; (B) low-energy mass spectrum; and (C) high-energy mass spectrum. Both spectra correspond to the time window of 8.7484 ± 0.0226 min; Figure S2. Chromatogram and mass spectra associated with the tentatively annotated pirimiphos-methyl candidate: (A) chromatogram of the [M+NH_4_]^+^ channel; (B) low-energy mass spectrum; and (C) high-energy mass spectrum. Both spectra correspond to the time window of 9.6187 ± 0.0232 min; Figure S3. Chromatogram and mass spectra associated with the tentatively annotated fenazaquin candidate: (A) chromatogram of the [M+Na]^+^ channel; (B) low-energy mass spectrum; and (C) high-energy mass spectrum. Both spectra correspond to the time window of 11.3238 ± 0.0232 min; Figure S4. Chromatogram and mass spectra associated with the tentatively annotated butoxycarboxim candidate: (A) chromatogram of the [M+NH_4_]^+^ channel; (B) low-energy mass spectrum; and (C) high-energy mass spectrum. Both spectra correspond to the time window of 2.0159 ± 0.0237 min; Figure S5. Chromatogram and mass spectra acquired within the retention-time window of the signal annotated by UNIFI as a pendimethalin candidate: (A) chromatogram of the [M+NH_4_]^+^ channel; (B) low-energy mass spectrum; and (C) high-energy mass spectrum. Both spectra correspond to the time window of 11.1250 ± 0.0232 min. The displayed high-energy ions have not been established as pendimethalin fragments; Figure S6. Chromatogram and mass spectra acquired within the retention-time window of the signal annotated by UNIFI as a silthiofam candidate: (A) chromatogram of the [M+H]^+^ channel; (B) low-energy mass spectrum; and (C) high-energy mass spectrum. Both spectra correspond to the time window of 8.8019 ± 0.0233 min. The displayed high-energy ions have not been established as silthiofam fragments; Figure S7. Chromatogram and mass spectra associated with the tentatively annotated esprocarb candidate: (A) chromatogram of the [M+NH_4_]^+^ channel; (B) low-energy mass spectrum; and (C) high-energy mass spectrum. Both spectra correspond to the time window of 11.7599 ± 0.0224 min.
