# Supplementary material for: Maternal Pharmaceutical Intake, Tobacco Smoking, and Environmental Pesticide Exposures: High-Resolution Serum Pesticide Screening, Longitudinal Fetal Ultrasound Biometry, and Postnatal Anogenital Distance in a Brazilian Cohort
Source: Biology (Basel). 2026 Oct 16;15(20):1869. doi: 10.3390/biology15201869 (PMC13701402; doi:10.3390/biology15201869)
Supplement: Supplementary file 1 [file biology-15-01869-s001.zip › biology-4617987-supplementary.pdf]

Supplementary Material - HRMS screening and spectral evidence for selected pesticide candidates.

Table S1. Analytical evidence and provisional identification confidence for the TEBUTAM candidate.

Parameter	Result / interpretation
Compound	Tebutam
Candidate molecular formula	Not reported
Theoretical / observed neutral mass (Da)	233.17796 / 233.1777
Observed m/z / adduct	234.1849 / $[M+H]^+$
Mass error (ppm)	-1.3
Expected / observed RT (min)	9.21 / 8.75
Retention-time difference (min)	-0.46 (observed − expected)
Fragment evidence	2 theoretical fragments found; 2 of 2 expected fragments found, as reported by UNIFI. Fragment m/z values are not provided.
Alternate assignments	NO (UNIFI field; alternatives not detailed)
MS/MS-related evidence	The spreadsheet provides fragment counts only; spectral agreement and precursor–fragment association cannot be assessed.
Identification confidence	Provisional Schymanski Level 2

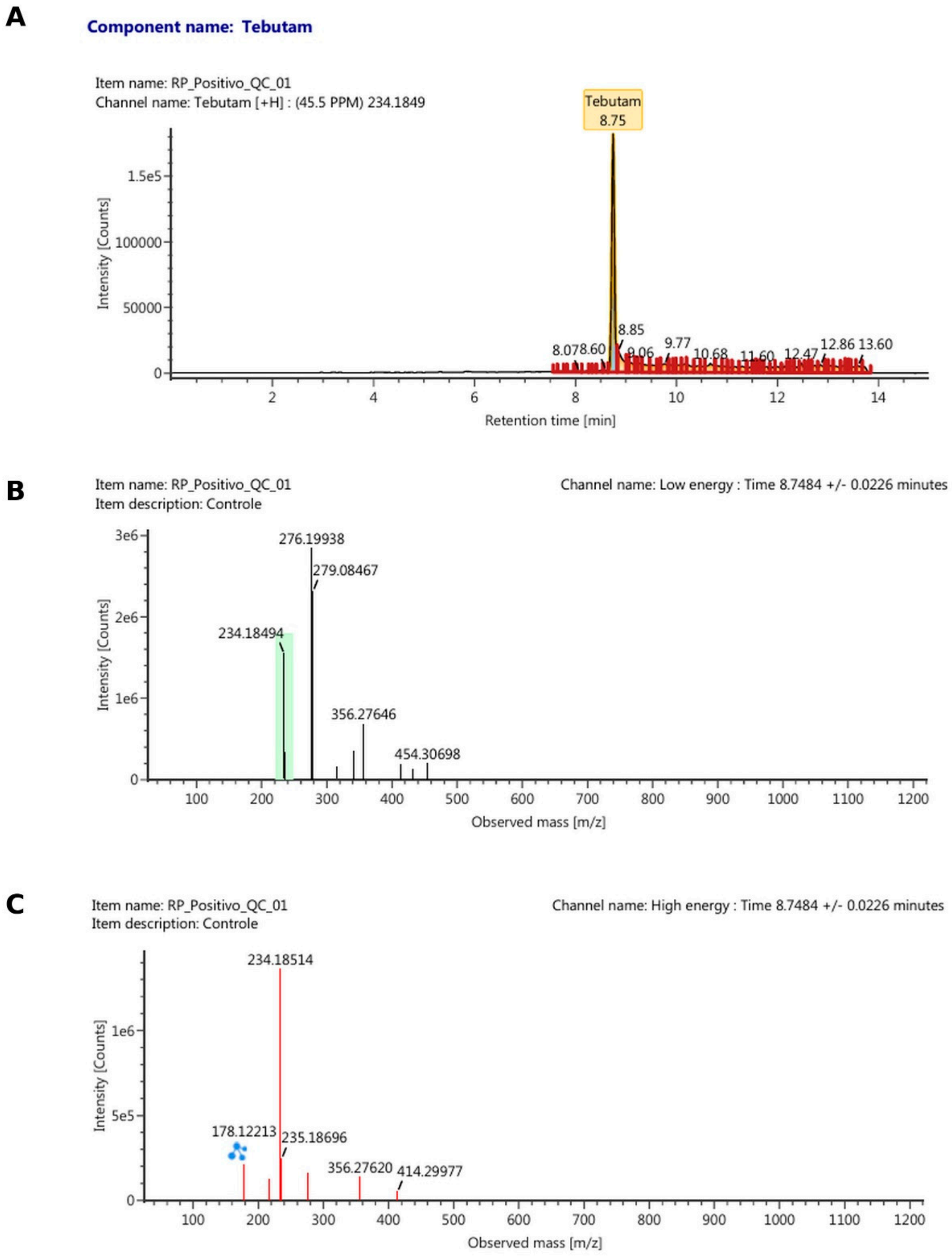

Figure S1. Chromatogram and mass spectra associated with the tentatively identified Tebutam candidate: (A) chromatogram of the $[M+H]^+$ channel, showing a peak at 8.75 min; (B) low-energy mass spectrum; and (C) high-energy mass spectrum. Both spectra correspond to the time window of 8.7484 ± 0.0226 min.

Table S2. Analytical evidence and provisional identification confidence for the PIRIMIPHOS-METHYL candidate.

Parameter	Result / interpretation
Compound (spreadsheet / image)	Pirimiphos-methyl / Pirimiphos-methyl
Molecular formula	Not reported
Theoretical / observed neutral mass (Da; spreadsheet)	305.09630 / 305.0962
Observed m/z / adduct (spreadsheet)	323.1301 / +NH4
Extraction-channel m/z (image)	323.1306; not independently established as measured precursor m/z
Mass error (ppm; spreadsheet)	-0.2
Expected / observed RT (min; spreadsheet)	9.91 / 9.63
Spectrum time window (min; image)	9.6187 ± 0.0232
Fragment evidence (spreadsheet)	6 theoretical fragments found; 1 / 2 expected fragments found.
Labelled high-energy ions (m/z)	119.08519; 135.08058; 281.13644; 415.21264; 485.21711. Not assigned to the candidate.
MS/MS-related evidence	Low- and high-energy spectra are supplied for the same stated time window.
Identification confidence	Provisional Schymanski Level 2

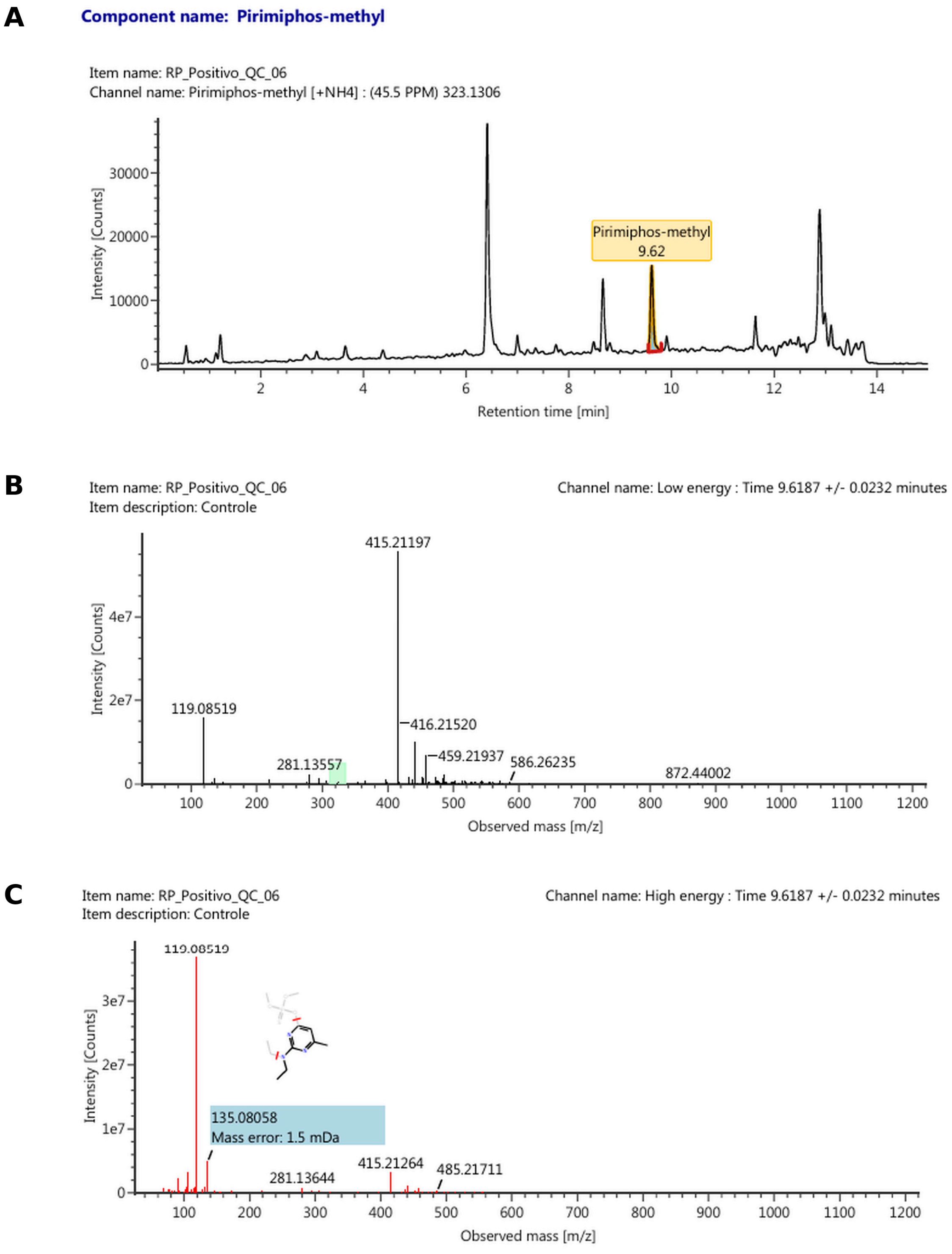

Figure S2. Chromatogram and mass spectra associated with the tentatively annotated pirimiphos-methyl candidate: (A) chromatogram of the $[M+NH_4]^+$ channel; (B) low-energy mass spectrum; and (C) high-energy mass spectrum. Both spectra correspond to the time window of 9.6187 ± 0.0232 min.

Table S3. Analytical evidence and provisional identification confidence for the FENAZAQUIN candidate.

Parameter	Result / interpretation
Compound (spreadsheet / image)	Fenazaquin / Fenazaquin
Molecular formula	Not reported
Theoretical / observed neutral mass (Da; spreadsheet)	306.17321 / 306.1736
Observed m/z / adduct (spreadsheet)	329.1629 / +Na
Extraction-channel m/z (image)	329.1629; not independently established as measured precursor m/z
Mass error (ppm; spreadsheet)	1.3
Expected / observed RT (min; spreadsheet)	11.46 / 11.32
Spectrum time window (min; image)	11.3238 ± 0.0232
Fragment evidence (spreadsheet)	1 theoretical fragments found; 1 / 4 expected fragments found.
Labelled high-energy ions (m/z)	149.02341; 150.02648; 329.17197; 451.36311; 575.27066. Not assigned to the candidate.
MS/MS-related evidence	Low- and high-energy spectra are supplied for the same stated time window.
Identification confidence	Provisional Schymanski Level 2

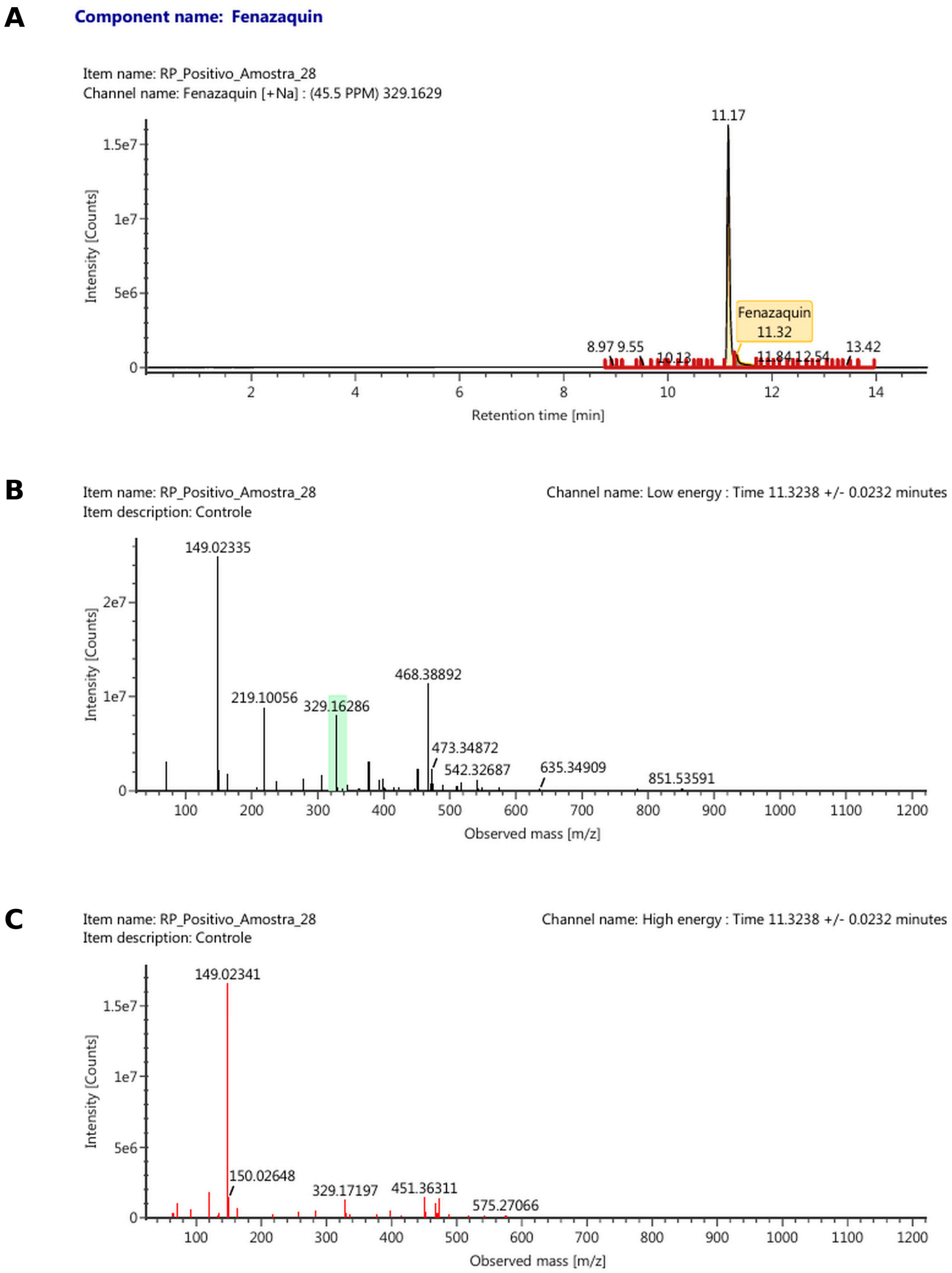

Figure S3. Chromatogram and mass spectra associated with the tentatively annotated fenazaquin candidate: (A) chromatogram of the $[M+Na]^+$ channel; (B) low-energy mass spectrum; and (C) high-energy mass spectrum. Both spectra correspond to the time window of 11.3238 ± 0.0232 min.

Table S4. Analytical evidence and provisional identification confidence for the BUTOXYCARBOXIM candidate.

Parameter	Result / interpretation
Compound (spreadsheet / image)	Butoxycarboxim / Butoxycarboxim
Molecular formula	Not reported
Theoretical / observed neutral mass (Da; spreadsheet)	222.06743 / 222.0679
Observed m/z / adduct (spreadsheet)	240.1017 / +NH4
Extraction-channel m/z (image)	240.1017; not independently established as measured precursor m/z
Mass error (ppm; spreadsheet)	1.8
Expected / observed RT (min; spreadsheet)	2.74 / 2.02
Spectrum time window (min; image)	2.0159 ± 0.0237
Fragment evidence (spreadsheet)	1 theoretical fragments found; 1 / 2 expected fragments found.
Labelled high-energy ions (m/z)	120.07739; 166.07891; 240.10194; 740.27386; 1091.43183. Not assigned to the candidate.
MS/MS-related evidence	Low- and high-energy spectra are supplied for the same stated time window.
Identification confidence	Provisional Schymanski Level 2

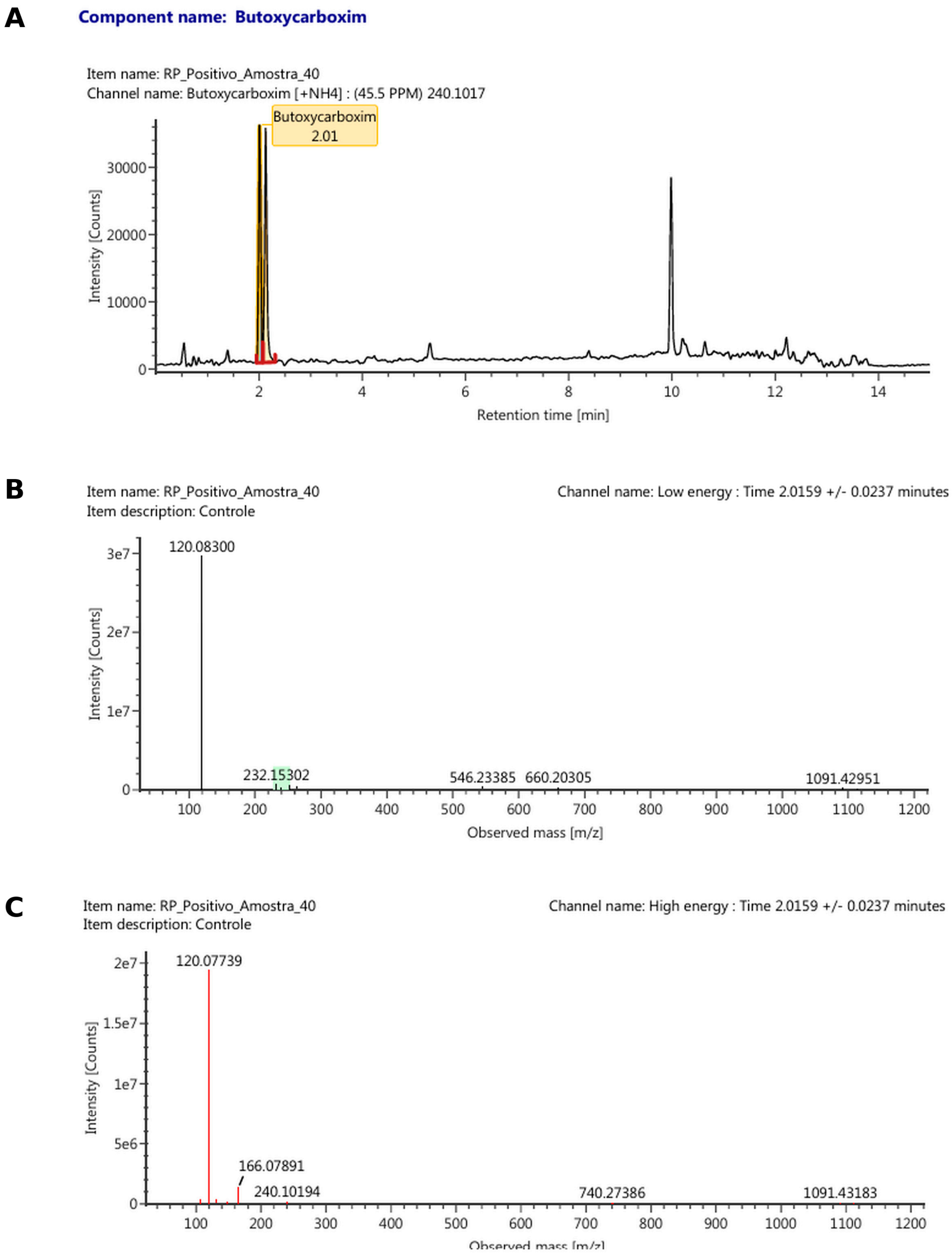

Figure S4. Chromatogram and mass spectra associated with the tentatively annotated butoxycarboxim candidate: (A) chromatogram of the $[M+NH_4]^+$ channel; (B) low-energy mass spectrum; and (C) high-energy mass spectrum. Both spectra correspond to the time window of 2.0159 ± 0.0237 min.

Table S5. Analytical evidence and provisional identification confidence for the PENDIMETHALIN candidate.

Parameter	Result / interpretation
Compound (spreadsheet / image)	Pendimethalin / Pendimethalin
Molecular formula	Not reported
Theoretical / observed neutral mass (Da; spreadsheet)	281.13756 / 281.1373
Observed m/z / adduct (spreadsheet)	299.1711 / NH4
Extraction-channel m/z (image)	299.1711; not independently established as measured precursor m/z
Mass error (ppm; spreadsheet)	-0.9
Expected / observed RT (min; spreadsheet)	10.83 / 11.12
Spectrum time window (min; image)	11.1250 ± 0.0232
Fragment evidence (spreadsheet)	1 theoretical fragments found; 1 / 1 expected fragments found.
Labelled high-energy ions (m/z)	119.08524; 184.07272; 297.23945; 415.14062; 485.30623; 788.62103; 794.60696; 796.61197. Not assigned to the candidate.
MS/MS-related evidence	Low- and high-energy spectra are supplied for the same stated time window.
Identification confidence	Provisional Schymanski Level 2

A

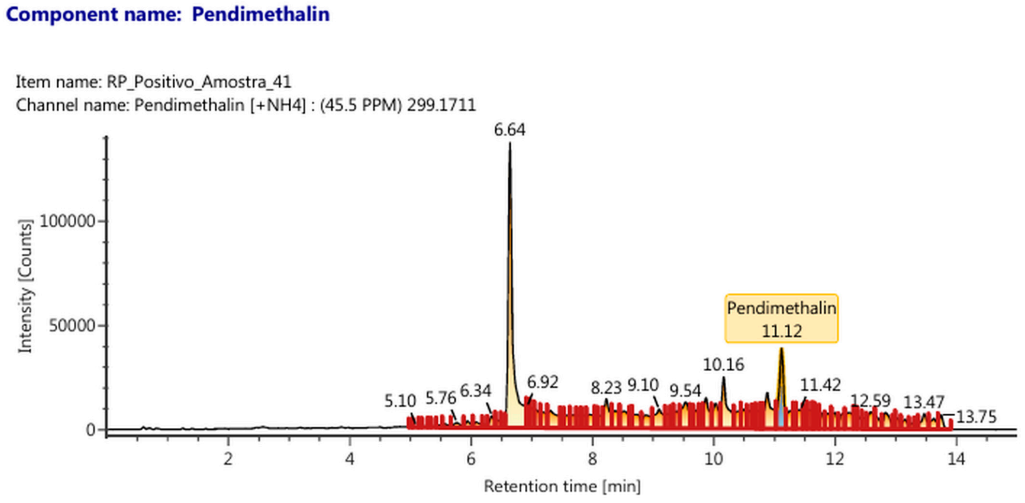

B

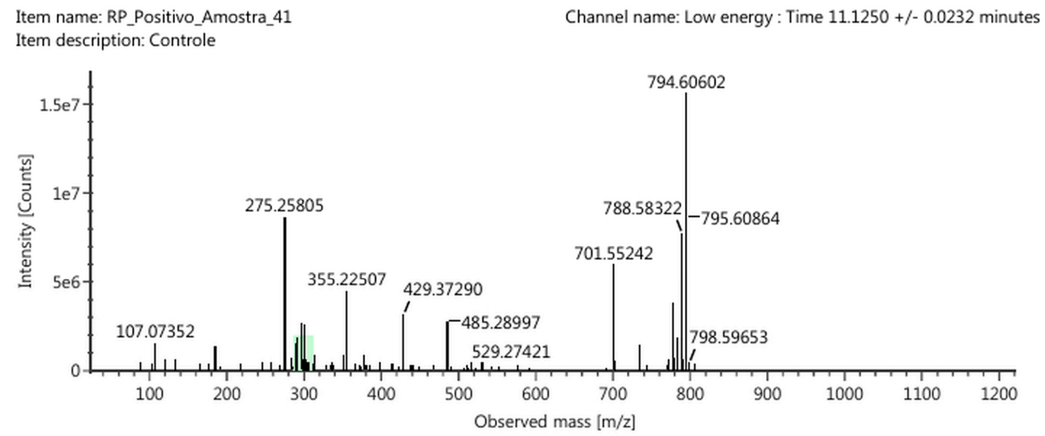

C

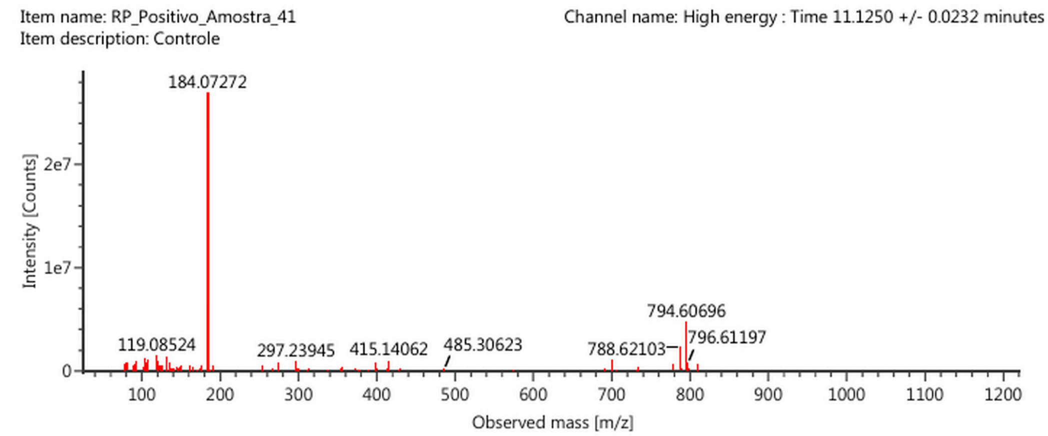

Figure S5. Chromatogram and mass spectra acquired within the retention-time window of the signal annotated by UNIFI as a pendimethalin candidate: (A) chromatogram of the $[M+NH_4]^+$ channel; (B) low-energy mass spectrum; and (C) high-energy mass spectrum. Both spectra correspond to the time window of 11.1250 ± 0.0232 min. The displayed high-energy ions have not been established as pendimethalin fragments.

Table S6. Analytical evidence and provisional identification confidence for the SILTHIOFAM candidate.

Parameter	Result / interpretation
Compound (spreadsheet / image)	Silthiofam / Silthiofam
Molecular formula	Not reported
Theoretical / observed neutral mass (Da; spreadsheet)	267.11131 / 267.1109
Observed m/z / adduct (spreadsheet)	268.1182 / +H
Extraction-channel m/z (image)	268.1181; not independently established as measured precursor m/z
Mass error (ppm; spreadsheet)	-1.4
Expected / observed RT (min; spreadsheet)	9.40 / 8.80
Spectrum time window (min; image)	8.8019 ± 0.0233
Fragment evidence (spreadsheet)	1 theoretical fragments found; 1 / 3 expected fragments found.
Labelled high-energy ions (m/z)	105.07035; 121.06392; 267.12341; 387.18188; 457.18548; 807.34047. Not assigned to the candidate.
MS/MS-related evidence	Low- and high-energy spectra are supplied for the same stated time window.
Identification confidence	Provisional Schymanski Level 2

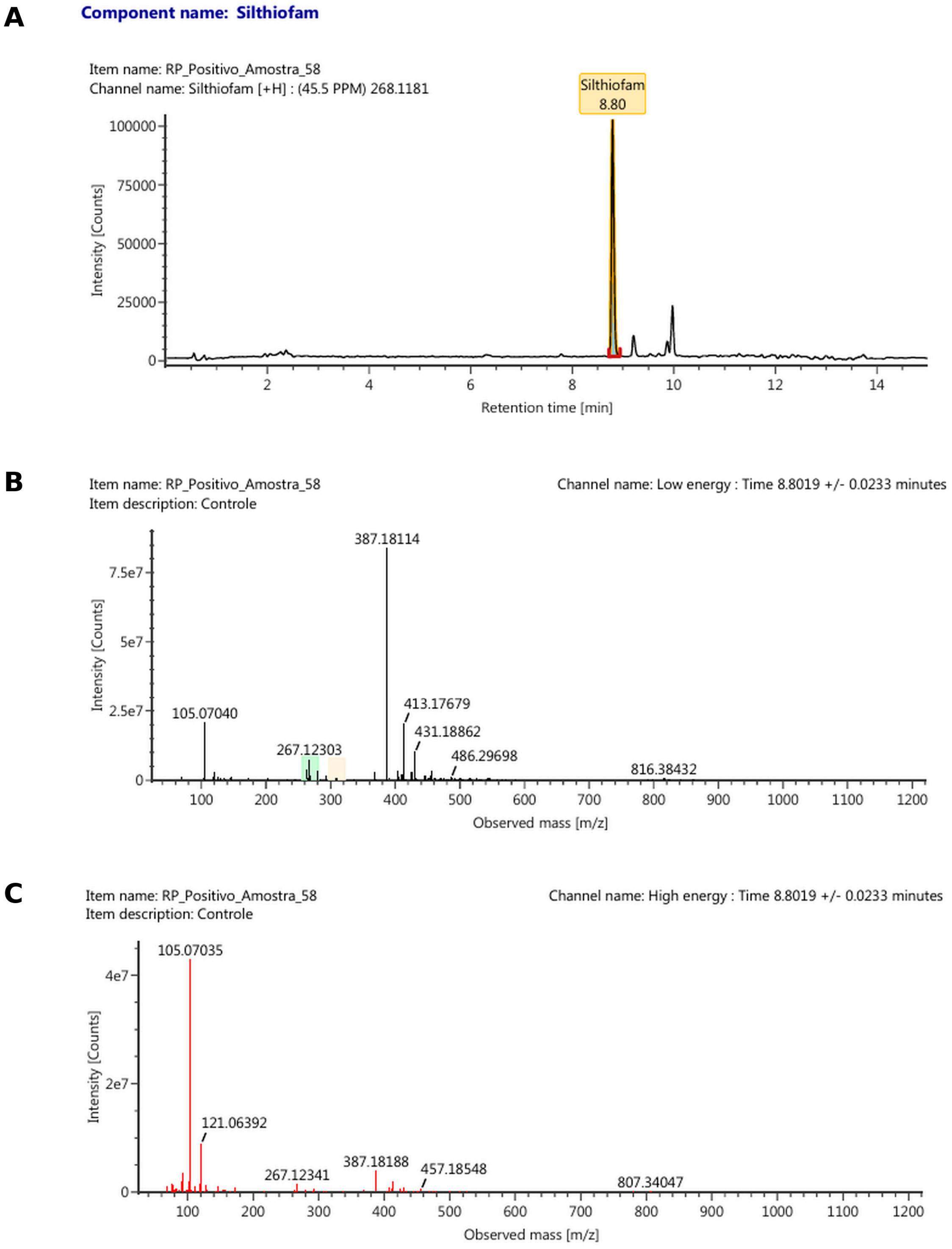

Figure S6. Chromatogram and mass spectra acquired within the retention-time window of the signal annotated by UNIFI as a silthiofam candidate: (A) chromatogram of the $[M+H]^+$ channel; (B) low-energy mass spectrum; and (C) high-energy mass spectrum. Both spectra correspond to the time window of 8.8019 ± 0.0233 min. The displayed high-energy ions have not been established as silthiofam fragments.

Table S7. Analytical evidence and provisional identification confidence for the ESPROCARB candidate.

Parameter	Result / interpretation
Compound (spreadsheet / image)	Esprocarb / Esprocarb
Molecular formula	Not reported
Theoretical / observed neutral mass (Da; spreadsheet)	265.15004 / 265.1502
Observed m/z / adduct (spreadsheet)	283.184 / NH4
Extraction-channel m/z (image)	283.1839; not independently established as measured precursor m/z
Mass error (ppm; spreadsheet)	0.6
Expected / observed RT (min; spreadsheet)	10.61 / 11.76
Spectrum time window (min; image)	11.7599 ± 0.0224
Fragment evidence (spreadsheet)	5 theoretical fragments found; 1 / 1 expected fragments found.
Labelled high-energy ions (m/z)	149.02350; 159.08134; 279.08307; 482.32542; 526.33972; 544.34018; 568.34074; 570.34292; 702.57265; 760.58642; 834.60299; 835.60583; 836.61434; 1020.75050; 1104.69032. Not assigned to the candidate.
MS/MS-related evidence	Low- and high-energy spectra are supplied for the same stated time window.
Identification confidence	Provisional Schymanski Level 2

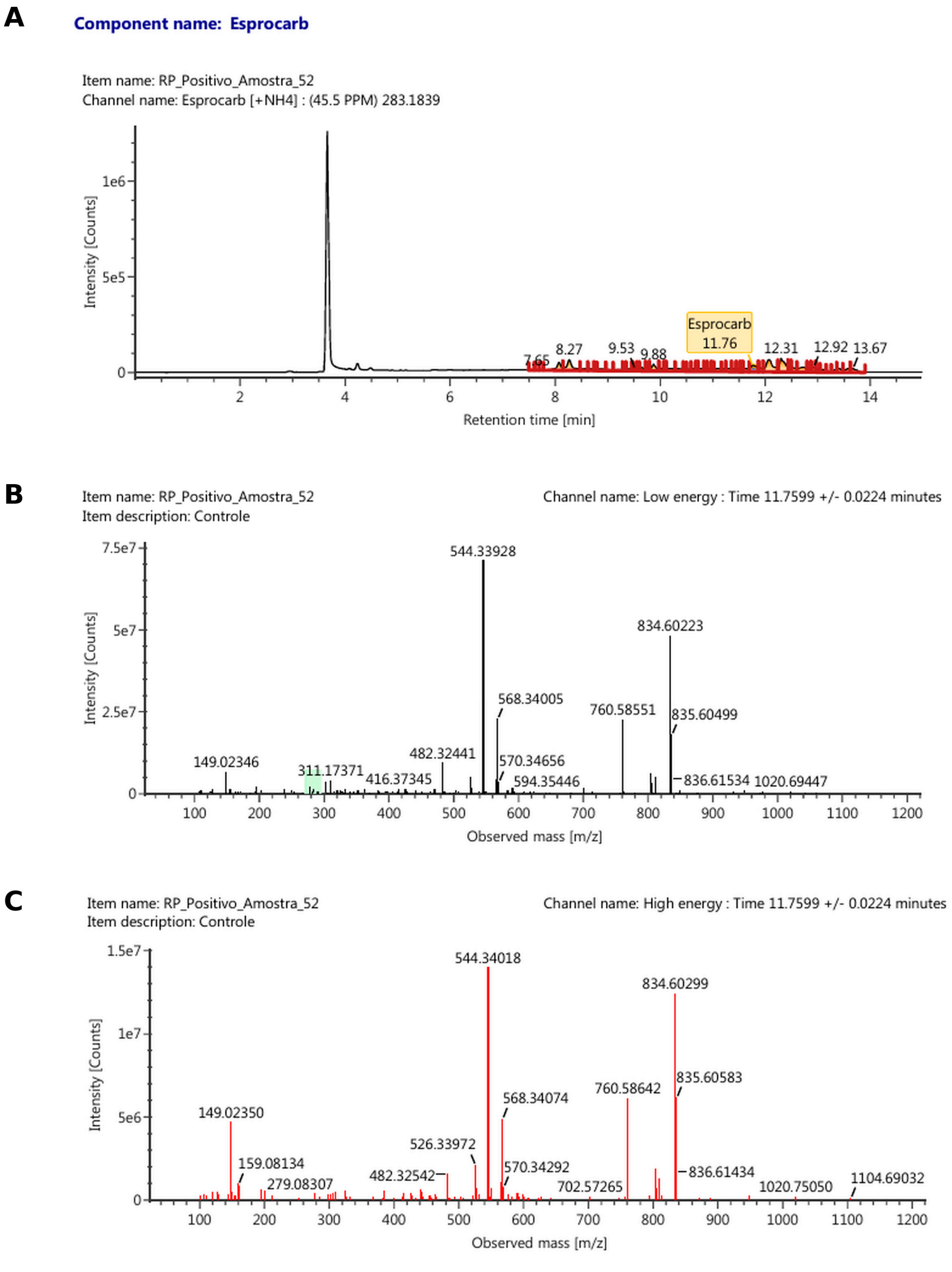

Figure S7. Chromatogram and mass spectra associated with the tentatively annotated esprocarb candidate: (A) chromatogram of the $[M+NH_4]^+$ channel; (B) low-energy mass spectrum; and (C) high-energy mass spectrum. Both spectra correspond to the time window of 11.7599 ± 0.0224 min.
